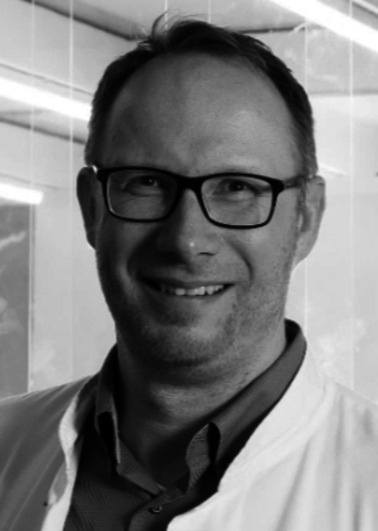# Antibiotic‐Derived Radiotracers for Positron Emission Tomography: Nuclear or “Unclear” Infection Imaging?

**DOI:** 10.1002/anie.202204955

**Published:** 2022-09-20

**Authors:** Arno Christiaan Gouws, Hendrik Gerhardus Kruger, Olivier Gheysens, Jan Rijn Zeevaart, Thavendran Govender, Tricia Naicker, Thomas Ebenhan

**Affiliations:** ^1^ Catalysis and Peptide Research Unit University of KwaZulu-Natal Durban 4000 South Africa; ^2^ Department of Nuclear Medicine Cliniques Universitaires Saint-Luc, and Institute of Clinical and Experimental Research Université Catholique de Louvain Brussels Belgium; ^3^ Nuclear Medicine Research Infrastructure NPC Pretoria 0001 South Africa; ^4^ Radiochemistry The South African Nuclear Energy Corporation Brits 0420 South Africa; ^5^ Preclinical Drug Development Platform North West University Potchefstroom 2520 South Africa; ^6^ Department of Chemistry University of Zululand KwaDlangezwa 3886 South Africa; ^7^ Department of Nuclear Medicine University of Pretoria Pretoria 0001 South Africa

**Keywords:** antibiotic-derived PET, imaging of infection, positron emission tomography, radiolabeling, radiotracers

## Abstract

The excellent features of non‐invasive molecular imaging, its progressive technology (real‐time, whole‐body imaging and quantification), and global impact by a growing infrastructure for positron emission tomography (PET) scanners are encouraging prospects to investigate new concepts, which could transform clinical care of complex infectious diseases. Researchers are aiming towards the extension beyond the routinely available radiopharmaceuticals and are looking for more effective tools that interact directly with causative pathogens. We reviewed and critically evaluated (challenges or pitfalls) antibiotic‐derived PET radiopharmaceutical development efforts aimed at infection imaging. We considered both radiotracer development for infection imaging and radio‐antibiotic PET imaging supplementing other tools for pharmacologic drug characterization; overall, a total of 20 original PET radiotracers derived from eleven approved antibiotics.

## Graphical abstract

This Review provides summary and critical evaluation, including the challenges and pitfalls, of antibiotic‐derived positron emission tomographic radiopharmaceutical development efforts aimed at infection imaging either considered for radiotracer development for infection imaging or radio‐antibiotic PET imaging supplementing other tools for pharmacologic drug characterization.

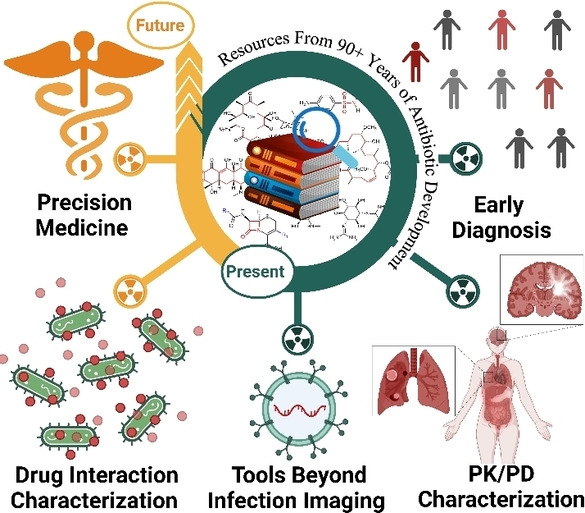

## 1 Introduction

Despite significant advances and increased availability of antimicrobial therapies, bacterial infections and the emergence of antimicrobial resistance persist as a worldwide health problem, causing significant morbidity and mortality.^[1]^ Accurate and early diagnosis of infections is of utmost importance for patient management and therapeutic decisions. However, the diagnosis of an infection can be challenging but imaging studies are often used for this and even to determine the extent of the infection.

Over the past decades, the use of nuclear imaging techniques for diagnosing infectious diseases has rapidly expanded with positron emission tomography (PET) being the most used method. The uptake mechanisms of current clinically available radiopharmaceuticals such as radiolabeled white blood cells (WBC), [^18^F]fluorodeoxyglucose ([^18^F]FDG), radiolabeled antibodies, or nanoparticles are not based on a direct interaction with bacterial pathogens, but on secondary host‐mediated inflammatory responses^[2]^ and are therefore not specific for infection. For instance, [^18^F]FDG‐PET accumulates in inflamed and malignant tissues based on a higher energy demand above neighboring tissues. While [^18^F]FDG‐PET has been shown to yield acceptable diagnostic accuracy in various infectious disorders, its diagnostic value is limited by its non‐specific accumulation mechanism, which has been well described.^[3]^ To overcome these limitations whilst coping with the ever‐increasing threat of (drug‐resistant) bacterial pathogens globally, research efforts are expanding to the development of more infection‐specific radiopharmaceutical tools based on direct interactions with infectious pathogens. This includes improving the overall diagnostic capabilities concerning specificities over inflammation or tumor growth whilst increasing sensitivity and resolution for the clinical setting. In the past decade, moderate progress has been made in discovering and evaluating promising novel candidates, which can be classified into synthetics and biomimetics; metabolic tracers; antibodies; antimicrobial peptides; and antibiotic‐derived tracers.[^4^, ^5^] Most candidates are currently under pre‐clinical evaluation with only some undergoing clinical trials

Historically, antibiotics have been extensively pursued as infection‐imaging vectors due to their well‐defined bactericidal or bacteriostatic mechanisms of action (MoA). This generally entails disrupting and inhibiting essential bacteriological processes through direct binding to participating molecular structures/enzymes with high affinity and selectivity—an important prerequisite for bacterial‐specific radiotracer development. Additionally, most antibiotics can be chemically classified as small organic compounds, which are of high value for radiochemical development using medicinal radioisotopes, especially diagnostic PET radionuclides that ideally match the physiological half‐life of the respective antibiotics.[^6^, ^7^] Although some structural changes may be tolerable, they are mostly limited to the non‐targeting entity of the antibiotic; common strategies are either the direct radiolabeling of the antibiotic with a radioisotope or the attachment of a radioisotope via a linker or chelator conjugate.^[8]^ This offers significant advantages over the more routinely used radiolabeling approaches that employ technetium‐99m, which can affect the validity of the imaging agents due to: *i) bactericidal behavior of unlabeled antibiotic (due to the larger antibiotic quantities required for a successful radiosynthesis); ii) complicated radiolabeling procedures with variable radiochemical purities; iii) complexation stability issues; and iv) the risk of modifications or radiometal complexation affecting pharmacokinetic/pharmacodynamic (PK/PD) parameters*.[^8^, ^9^]

**Figure 1:**
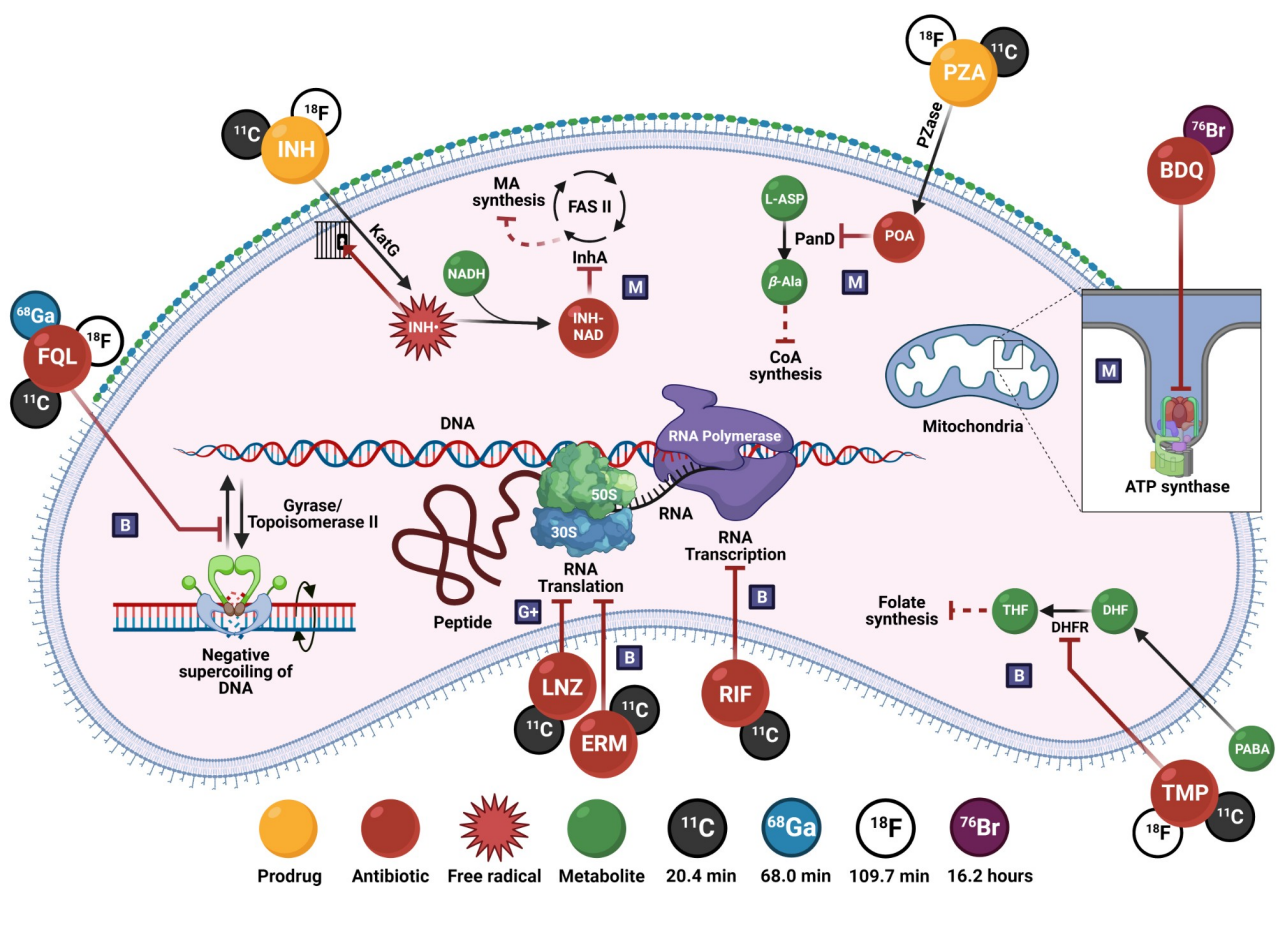
PET radio‐antibiotics evaluated as infection imaging agents and their respective cellular targets. Abbreviated content: G+: Gram‐positive specific; M: Mycobacteria‐specific; B: broad spectrum; FQL: fluoroquinolones; TMP: trimethoprim; INH: isoniazid (including PT70 and PT119); PZA: pyrazinamide; RIF: rifampin; LNZ: linezolid; BDQ: bedaquiline; ERM: erythromycin; DHFR: dihydrofolate reductase; KatG: mycobacterial catalase–peroxidase; InhA: enoyl‐ACP reductase; FAS II: type II fatty acid synthase system; Pzase: pyrazinamidase; PanD: aspartate 1‐decarboxylase; PABA: *p*‐aminobenzoic acid; DHF: dihydrofolate; THF: tetrahydrofolate; NADH: nicotinamide adenine dinucleotide; L‐ASP: L‐aspartic acid; β‐Ala: β‐alanine; CoA: co‐enzyme A; ATP: adenosine triphosphate. Created with Biorender.com.

This Review provides a comprehensive summary of the relevant antibiotic‐derived PET radiotracers, either grouped by radiotracer development for infection imaging, or radio‐antibiotic PET imaging for pharmacologic drug characterization. Figure 1 illustrates the different PET radio‐antibiotics including their respective MoA. Profound results were achieved so far, however tracer translation to clinical settings seems complex and lengthy. Therefore, a critical in‐depth evaluation addressing the challenges and pitfalls of developing antibiotic‐derived PET radiotracers as infection imaging agents is provided. Radiolabeled metronidazole and puromycin have been excluded due to their predominant use outside the realms of infection imaging, since they are employed as markers for tumor hypoxia^[10]^ and protein synthesis rate,^[11]^ respectively. The use of radiolabeled antibiotics for single‐photon emission computerized tomography (SPECT) and non‐antibiotic PET/SPECT radiotracers has been extensively reviewed elsewhere.[^4^, ^12^, ^13^, ^14^]

## 2 Antibiotics as PET Imaging Agents

### 2.1 Antifolates (Trimethoprim)

Trimethoprim (TMP) is a broad‐spectrum antibiotic prescribed together with sulfamethoxazole to treat a variety of commonly‐encountered infections.^[15]^ Trimethoprim exerts its antimicrobial activity by blocking bacterial folate activation through inhibition of dihydrofolate reductase (DHFR). Although mammals generally display an analogue of the DHFR enzyme, the substrate‐binding site is significantly different from its bacterial counterpart, which results in a significantly lower TMP binding affinity.^[16]^ For instance, the half maximal inhibitory concentration (IC_50_) of TMP for human DHFR is 490 μM (inhibitor constant, *K*_i_=3700 nM), versus 7 nM (*K*_i_=1.3 nM) for *E. coli dhfr*.^[17]^ Hence, Sellmyer et al. synthesized [^11^C]trimethoprim and [^18^F]fluoropropyl‐trimethoprim and evaluated their potential as bacterial‐specific imaging agents.[^18^, ^19^, ^20^]

#### 2.1.1 [^11^C]Trimethoprim ([^11^C]TMP)

[^11^C]TMP was radiosynthesized by trapping [^11^C]CH_3_I (from cyclotron‐produced [^11^C]CO_2_) in a reaction mixture of DMF, base (aqueous NaOH), and a TMP−OH precursor. The reaction mixture was heated to 70 °C and allowed to react for five minutes, followed by purification with reverse‐phase HPLC. A >98 % purity of [^11^C]TMP was obtained in 30 minutes (specific activity: 18.5–37.0 GBq μmol^−1^; yield: 50–60 %).[^20^, ^21^]

A dot blot assay with various concentrations of *E. coli* DHFR incubated with [^11^C]TMP showed a concentration‐dependent, specific radiotracer binding.^[21]^ To assess bacterial [^11^C]TMP uptake and determine if antibiotic resistance may affect radiotracer uptake levels, extensive in vitro uptake studies were performed across a panel of wild‐type‐ and TMP‐resistant bacterial strains expressing mutant DHFR enzymes.^[20]^ In all cases, both drug‐susceptible and ‐resistant bacterial strains showed similar and sustained [^11^C]TMP uptake compared to negative controls (either DHFR knockout strain, heat‐killed, or TMP‐blocked bacteria). As the underlying accumulation mechanisms, resistant strains carried two or more DHFR expressing genes. The wildtype DHFR gene is maintained in conjunction with another DHFR gene that conveys resistance. Thus, this wildtype DHFR copy in TMP‐resistant strains is responsible for the sustained, high levels of intracellular [^11^C]TMP.^[20]^

The biodistribution of [^11^C]TMP‐PET in healthy human subjects^[19]^ and a proof‐of‐concept study in patients with known or suspected bacterial infections^[20]^ showed hepatic tracer accumulation and activity in kidneys and bladder—representing TMP metabolism and excretion. A patient with biopsy‐proven methicillin‐sensitive *S. aureus* (MSSA) discitis, scanned with [^11^C]TMP, showed asymmetric uptake in the left L4‐5 facet that resolved completely on the follow‐up scan. Three patients with cystic fibrosis (CF) co‐registered focal uptake of [^11^C]TMP within some (but not all) lesions identified with computed tomography (CT). In comparison, a patient with [^18^F]FDG‐avid metastatic lung adenocarcinoma showed negligible [^11^C]TMP uptake.

#### 2.1.2 [^18^F]F‐Propyl‐Trimethoprim ([^18^F]FP‐TMP)

[^18^F]FP‐TMP was synthesized with excellent specific activity (185–555 GBq μmol^−1^), and radiochemical purity (99 %) by radio‐fluorination of a *p*‐propyl‐mesylate derivative of TMP.^[18]^ The unlabeled FP‐TMP showed similar specificity against *E. coli*, *S. aureus*, and *P. aeruginosa* as reported for TMP,[^17^, ^18^] indicating that derivatization of TMP had a minor effect on its DHFR binding capabilities, i.e., the *K*_d_ value of 1.3 nM (TMP) increased to 0.465 nM for [^18^F]FP‐TMP. [^18^F]FP‐TMP was significantly retained by *E. coli* and *S. aureus*, while *P. aeruginosa* (resistant to TMP) exhibited lower uptake and late retention. [^18^F]FP‐TMP‐PET imaging in a mouse model bearing pathological processes within musculoskeletal tissues (*E. coli* or *S. aureus* infection, sterile inflammation, and tumor) revealed significantly elevated radiotracer signals, exclusively in the infected muscles (target to non‐target ratio [T/NT] of 2.7).^[18]^ The [^18^F]FP‐TMP biodistribution was dominated by rapid renal excretion with favorably low radioactivity observed in other organs, except for higher retention in bone (marrow), which was also evident in non‐human primates ([^18^F]FP‐TMP binding to hematopoietic cells).

#### 2.1.3 Clinical Translation of Radiolabeled Trimethoprim

Clinical translation of radiolabeled trimethoprim, e.g., [^18^F]FP‐TMP‐ and [^11^C]TMP‐PET imaging, is significant proof that the development of novel antibiotic‐derived PET tracers is feasible. [^11^C]TMP has already proven to be selective in imaging of bacterial infections in patients with suspected infections. While antibiotic resistance can directly affect antibiotic‐based tracer uptake, [^11^C]TMP‐PET imaging has shown promising results in detecting TMP‐resistant and multi‐drug resistant clinical strains.[^18^, ^20^] The relatively low tracer uptake ([^18^F]FP‐TMP‐T/NT<3.0) in the target tissue) could, however, be the limiting factor in its clinical usefulness and is part of an ongoing clinical trial.^[22]^

### 2.2 Fluoroquinolones

Fluoroquinolones target a highly conserved bacterial type II topoisomerase (gyrase and topoisomerase IV), an enzyme, which regulates DNA folding by introducing positive and negative DNA supercoils.^[23]^ The MoA of fluoroquinolones involves the simultaneous intercalation of two drug molecules in between the topoisomerase IV–DNA complex after the introduction and breakage of double‐stranded DNA. The increase in enzyme–DNA cleavage complexes that prevent DNA re‐ligation leads to DNA fragmentation which, if enough, overwhelms the DNA repair mechanisms and leads to cell death. In the early 1990s, ^99m^Tc‐labeled ciprofloxacin was synthesized.^[24]^ The promising results made [^99m^Tc]Tc‐ciprofloxacin commercially available (*Infecton®*), and it was used for clinical imaging of infectious diseases.^[25]^ However, subsequent studies were hampered by a lack of specificity, which led to its discontinuation.^[26]^ As an alternative to [^99m^Tc]Tc‐ciprofloxacin, other fluoroquinolone derivatives (including ciprofloxacin analogues) have been radiolabeled with PET‐radioisotopes.[^27^, ^28^, ^29^, ^30^, ^31^, ^32^, ^33^, ^34^]

#### 2.2.1 [^18^F]F‐Ciprofloxacin

In 2003, Langer et al. developed a radiosynthesis method for [^18^F]F‐ciprofloxacin, without altering the original structure of the drug, by substituting intrinsic fluorine with a radioactive fluorine‐18. This method yielded radiochemically‐pure product for initial human imaging studies; however, only a low radiochemical yield (RCY) of 2.5 % and highly variable, low specific activities (433±203 MBq μmol^−1^) were achieved.[^32^, ^33^, ^34^]

In vitro, [^18^F]F‐ciprofloxacin accumulates rapidly in ciprofloxacin‐susceptible *E. coli*, yet this accumulation was ≈90 % reversible (efflux) after phosphate‐buffered saline (PBS) washes. This tracer accumulation was not hindered by co‐incubation with increasing concentrations of unlabeled ciprofloxacin.^[34]^ The [^18^F]F‐ciprofloxacin PK/PD parameters studied in pre‐dosed healthy human volunteers showed that [^18^F]F‐ciprofloxacin rapidly spreads through all tissues, except the brain (lowest signal). The highest uptake was seen in the kidneys and liver with moderate levels of radioactivity being recognized in the myocardium, muscle tissue, spleen, and lungs. Tracer clearances from muscle and lung tissue were severely delayed (half‐life, *t*_1/2_>130 minutes) when compared to the other compartments (*t*_1/2_<13 minutes).^[33]^ [^18^F]F‐ciprofloxacin‐PET imaging performed in four patients with clinically proven ciprofloxacin‐susceptible soft‐tissue infections showed rapid and localized tracer signal at the affected areas. While it was easy to identify the infection site, the radiotracer signal cleared from the target tissue at the same rate as in healthy tissue (similarly to [^99m^Tc]ciprofloxacin, a pharmacokinetic that is based on increased blood flow and vascular permeability at the infection site).^[34]^

#### 2.2.2 N4′‐3‐[^18^F]F‐Propyl‐Ciprofloxacin ([^18^F]16)

Guided by structure–activity relationship analyses for ciprofloxacin, Sachin et al. (2014) followed a labeling strategy that involved N4′‐substituted ciprofloxacin derivatives, which finally resulted in the total synthesis of a fluorine‐18 radiolabeled fluoropropyl‐ciprofloxacin analogue (denoted as [^18^F]16).^[27]^ The radiolabeling solution used a carrier‐free [^18^F]fluoride displacement reaction of the N4′‐3‐methanesulfonyl‐propyl‐ciprofloxacin methyl ester with *N*‐*tert*‐Bu−N[^18^F]F. The synthesis procedure yielded [^18^F]16 with high radiochemical purity (>99 %) and excellent specific activity (149±75 GBq μmol^−1^) within 100 minutes from the end of bombardments. (RCY=40 %; decay corrected). In vitro experiments with cold [F]16 showed the most efficient antibacterial activity against *E. coli TOP10* (minimum inhibitory concentration) MIC_50_: 3.1±0.1 ng mL^−1^), compared to *E. coli DH5* (MIC_50_: 157±4.0 ng mL^−1^); and it was capable of inhibiting *E. coli* DNA gyrase (IC_50_: 9.6±3.6 μg mL^−1^). In addition, co‐incubation with 25 nmol of a non‐radioactive analogue decreased uptake by 59 %, thereby confirming specific binding.^[27]^ To date, no follow‐up studies have been published.

#### 2.2.3 [^68^Ga]Ga‐Ciprofloxacin

In order to produce higher radiochemical yields than [^18^F]ciprofloxacin, Satpati et al. (2016) introduced a benzyl thiourea linker into ciprofloxacin to furnish the derivatives [^68^Ga]Ga‐*p*‐SCN‐Bz‐NOTA and [^68^Ga]Ga‐*p*‐SCN‐Bz‐DOTA (denoted [^68^Ga]‐1 and [^68^Ga]‐2, respectively).^[28]^ Both radiotracers were successfully radiolabeled with ^68^Ga with a final RCY>90 % and specific activities of 6.2±0.4 GBq μmol^−1^. Significant amounts of the tracer were retained by viable *S. aureus* cell cultures (0.9–1.0 % and 1.6–2.3 % for the NOTA and DOTA analogues, respectively), when compared to non‐viable cultures (<0.3 % uptake). However, retention could not be blocked by excess unlabeled ciprofloxacin (similar to [^99m^Tc]Tc‐/[^18^F]F‐ciprofloxacin), signifying non‐specific binding. Despite indications of non‐specific binding, [^68^Ga]‐1/2 was equally able to distinguish *S. aureus*‐infected tissue from healthy or inflamed tissue, displaying retained tracer activity at the infection site for up to 120 minutes in a rat myositis model. This is in direct contrast to what was observed with [^18^F]F‐/[^99m^Tc]Tc‐ciprofloxacin, where unsaturable,/non‐specific binding was thought to contribute to rapidly‐reversable tracer uptake by bacteria.^[28]^ The mismatch in these results may either be due to different in vitro test protocols, different specific radioactivity used in vivo and therefore PK dependent concentration effects, or adverse tissue pathology experienced in the animal model.

Alternatively, Koźmiński et al. used direct DOTA‐conjugation to ciprofloxacin ([^68^Ga]Ga‐DOTA‐ciprofloxacin with a radiochemical purity (RCP) of >90 %.^[35]^ In vitro results showed that after 60 minutes, *S. aureus* retained 1.1±0.2 % of the injected dose (ID) and *P. aeruginosa* 1.3±0.3 %ID; however, no negative controls were examined, but the results were rather compared to those of the [^68^Ga]‐1/2 in the literature.

#### 2.2.4 [^18^F]F‐Fleroxacin, [^18^F]F‐Trovafloxacin and [^18^F]F‐Lomefloxacin

Fischman et al. reported the successful radiosynthesis of [^18^F]F‐fleroxacin, the first PET‐compatible fluoroquinolone, in 1993, and [^18^F]F‐trovafloxacin in 1996.[^29^, ^30^] In subsequent studies, the same group used both radiotracers in conjunction with PET to quantify the in vivo and in situ biodistribution and behavior in a myriad of healthy and bacterial‐infected animal models (which included mice, rats and rabbits), healthy human volunteers, and patients with confirmed bacterial infections.[^36^, ^37^, ^38^, ^39^, ^40^] In 1996, Tewson et al. conducted similar in vivo biodistribution measurements of [^18^F]F‐lomefloxacin in a pig and healthy human volunteers, but through a less conventional oral administration instead of IV injection of the radiotracer bolus.^[31]^

Synthesis of radiochemically‐pure [^18^F]F‐fleroxacin (≈1.85 GBq μmol^−1^) was achieved over 90 minutes by the nucleophilic substitution of ^18^F to a reactive methylsulfonyl ester fleroxacin precursor, but this only provided 5–8 % RCY.^[29]^ [^18^F]F‐trovafloxacin and [^18^F]F‐lomefloxacin were radiosynthesized by ^18^F‐exchange with ^19^F in a reaction mixture containing the relevant antibiotic, Kryptofix 2.2.2, ^18^F‐fluoride and K_2_CO_3_ dissolved in DMSO and heated to 160 °C for 15 or 60 minutes, respectively. Subsequent HPLC purification of [^18^F]F‐trovafloxacin produced the radiochemically‐pure product (RCY 15–30 %; 45 minutes).^[30]^ On the other hand, [^18^F]F‐lomefloxacin was recrystallized by adding the reaction mixture to a boiling solution of 2 N HCl and ethanol (ratio of 8 : 3 ml) saturated with lomefloxacin, followed by cooling on ice for 20 minutes. This unconventional method produced radiochemically‐pure [^18^F]F‐lomefloxacin (RCY=20 %; 90 minutes).^[31]^ The specific activities for [^18^F]F‐trovafloxacin or [^18^F]F‐lomefloxacin were not reported.

PET and/or ex vivo measurements of [^18^F]F‐fleroxacin and [^18^F]F‐trovafloxacin biodistribution in healthy mice, rats, and human volunteers are reported.[^29^, ^30^, ^36^, ^37^, ^38^, ^39^, ^40^] In summary, the respective radiotracers showed rapid clearance from the blood (i.e., within 50 minutes in humans) and significant uptake was observed in all organs and peripheral tissues. Across all subjects, the peak radiotracer signals were determined for the kidneys, liver (gallbladder) and bowel, followed by moderate uptake in the myocardium, lungs, and spleen. Both radiotracers were generally renally excreted (approx. 60 %), with some hepatobiliary excretion occurring upon delayed metabolism. In addition, oral administration of [^18^F]F‐lomefloxacin behaved similarly to [^18^F]F‐fleroxacin and [^18^F]F‐trovafloxacin in healthy volunteers, but with a delay of 40 minutes due to the first‐pass effect.^[31]^ [^18^F]F‐fleroxacin and [^18^F]F‐trovafloxacin were further studied in rats and rabbits bearing a thigh infection of *E. coli* using PET image‐guided quantification.[^38^, ^40^] The peak radiotracer concentrations in infected tissues were variable between animal species. In rats for example, [^18^F]F‐fleroxacin accumulation was remarkably similar between healthy and infected tissue, while [^18^F]F‐trovafloxacin displayed significantly elevated concentrations in the infected versus healthy tissues (*P*<0.01). In rabbits, however, [^18^F]F‐trovafloxacin and [^18^F]F‐fleroxacin concentrations were slightly increased in infected tissues.

Furthermore, the PET‐image‐guided pharmacokinetics of [^18^F]F‐fleroxacin was studied in patients with acute exacerbations of chronic bronchitis and patients with complicated urinary tract infections (UTI).^[30]^ Three parameters were measured and compared between healthy and infected tissues; these included the peak and plateau concentrations and normalized area under the curve (AUC) values. For all pulmonary infections, these parameters were lower at sites of active infection: *P*<0.02, *P*<0.001, and *P*<0.005, respectively. In contrast, only the AUC values were elevated in the infected kidneys of patients with complicated UTIs. The authors attributed the discrepancy in these results to pulmonary fibrosis restricting drug penetration in the lungs, and elevated blood flow to the kidneys, along with radiotracer delivery due to inflammation caused by the infections. However, no further studies were done to test this hypothesis.

#### 2.2.5 Relevance and Limitations of Radiolabeled Fluoroquinolones

Ciprofloxacin was the first antibiotic explored as an infection‐specific imaging agent, mainly because of its broad‐spectrum antibiotic activity.^[41]^ Evidently, up to 2020, [^18^F]F‐ciprofloxacin was the only antibiotic‐derived PET probe that had undergone clinical trials as an infection‐specific radiotracer. It was only recently followed by [^18^F]FP‐TMP and [^11^C]TMP (see the previous section). Nonetheless, more recently performed radiolabeling approaches, such as those developed for [^68^Ga]‐1/2 and [^18^F]F16, have led to increased target‐specific binding in vitro and improved bacterial retention in vivo. However, neither has been translated clinically. The marked increase in bacterial‐specific accumulation and retention is surprising, since more prominent structural alterations to commercialized antibiotics tend to do the opposite. The contributing mechanisms behind this phenomenon are still unclear.

### 2.3 Isoniazid

Isoniazid, also known as isonicotinic acid hydrazide (INH), is an antibiotic prodrug with exclusive bactericidal activities towards mycobacterial species, due to its ability to disrupt mycolic acid biosynthesis, a crucial building block required for the generation and maintenance of the distinct membrane structure of mycobacteria. Briefly, INH diffuses passively across the cell membrane and undergoes oxidative activation by mycobacterial catalase–peroxidase (KatG), resulting in the formation of an isonicotinyl radical.^[42]^ Due to the lower intracellular pH levels maintained within mycobacterial cells, the isonicotinyl radical becomes intracellularly trapped.^[43]^ Upon intracellular oxidation, the isonicotinyl radical conjugates with nicotinamide adenine dinucleotide (NAD) to form an INH–NAD adduct, which is a competitive inhibitor of enoyl‐acyl carrier protein reductase (InhA), an essential enzyme required for mycolic acid biosynthesis.^[44]^ The combination of high affinity (*K*_i_=5.0 nM) and slow adduct dissociation time of the ternary INH‐NAD‐InhA complex (*t*_1/2_=43 minutes), together with metabolite trapping, and exclusive activity against mycobacteria, makes INH a promising candidate for development as a mycobacterial specific radiotracer.

#### 2.3.1 2‐[^18^F]F‐Isonicotinic Acid Hydrazide (2‐[^18^F]INH)

In 2002, Amartey et al. described the first radiosynthesis of 2‐[^18^F]INH and conducted initial in vitro bacterial accumulation assays and biodistribution experiments with a mouse infection model.[^45^, ^46^] However, these results were rendered insignificant by the use of irrelevant bacterial strains that have no reported susceptibility to INH (*S. pneumoniae* and *E. coli*). Surprisingly, however, the authors reported significant in vitro levels of radiotracer retention by *S. pneumoniae* and by *E. coli*. In 2012, Weinstein et al. adopted the above mentioned method to produce 2‐[^18^F]INH with relatively low specific activity (7.4  MBq μmol^−1^) and sufficient radiochemical purity (95 %) for biological evaluation as a potential mycobacteria‐specific imaging agent.^[47]^ 2‐[^18^F]INH was achieved through utilizing a nucleophilic ^18^F‐displacement reaction on an ethyl‐2‐(trimethylammonium)‐isonicotinate precursor. MIC calculation for cold 2‐F‐INH revealed that the structural modification required to incorporate fluorine‐18 resulted in reduced activity against wild‐type *M. tuberculosis* (MIC: 8.0 μg ml^−1^ vs. 0.025 μg ml^−1^). However, in vitro enzyme inhibition assays indicated that 2‐F‐INH still followed the MoA as INH.^[47]^ Accordingly, the authors reported that the wild‐type *M. tuberculosis* strain readily retains 2‐[^18^F]INH and retention was significantly reduced in a highly INH‐resistant KatG M1 A mutant strain of *M. tuberculosis*. The total uptake was not quantified. Lack of 2‐[^18^F]INH retention was also observed in heat‐killed bacterial suspensions, further corroborating the specificity of 2‐[^18^F]INH.

Weinstein et al. performed 2‐[^18^F]INH‐PET‐image‐guided biodistribution in BALB/c mice, which correlated well with data reported by Amartey et al.^[47]^ At the site of INH metabolism, significant hepatic tracer uptake and retention was seen, aside from avid renal excretion. Persistent, but lower tracer uptake was evident for the myocardium; hence, infections affecting the heart and liver might be difficult to visualize.^[47]^ However, the capability of 2‐[^18^F]INH‐PET/CT imaging of well‐defined pulmonary tuberculous lesions in C3HeB/FeJ mice was described.^[47]^ The 2‐[^18^F]INH T/NT ratio of 1.67±0.04 was quantified for infected pulmonary foci following imaging of C3HeB/FeJ mice at 90 minutes, which co‐registered well on the CT image, suggesting that 2‐[^18^F]INH can penetrate these fairly restricted tissues effectively. Most importantly, Weinstein et al. demonstrated the excellent blood–brain‐barrier (BBB) penetration properties of 2‐[^18^F]INH within 15 minutes—a very desirable aspect to eventually visualize tuberculosis (TB)‐derived meningitis in the clinical setting.^[47]^

#### 2.3.2 [^11^C]Isonicotinic Acid Hydrazide ([^11^C]INH)

In 2010, Liu et al. reported the successful synthesis of [^11^C]INH.^[48]^ The synthesis method features a reaction between ^11^C‐labeled hydrogen cyanide and iodopyridine, catalyzed by tetrakis(triphenylphosphine)palladium, to form a [^11^C]cyanopyridine intermediate. This reaction is followed by hydrolysis of the cyanide by hydrazine to form [^11^C]INH. An average yield above 45 % (decay‐corrected, calculated from [^11^C]HCN) was achieved (50 minutes; RCP≥99 %; 5.0 GBq μmol^−1^). Subsequent pharmacokinetic analysis using [^11^C]INH‐PET in healthy baboons indicated that [^11^C]INH and/or its metabolites rapidly distribute within the myocardium, lungs, liver and kidneys. However, the authors noted significantly less activity‐related drug concentration within the kidneys, liver and lungs, compared to rodents.[^49^, ^50^]

#### 2.3.3 Relevance and Limitations of Radiolabeled Isoniazid

[^99m^Tc]INH‐SPECT performed well in a phase I clinical trial as an *M. tuberculosis* lung and bone infection diagnosis tool in 20 patients with confirmed infection.^[51]^ Even though [^99m^Tc]INH was shown to be effective and feasible as a TB‐specific SPECT radiotracer, no further progress has been reported thus far. Data from this study revealed that ^99m^Tc‐radiolabeling INH altered several of its characteristics (unwanted uptake in intestines, gallbladder, lung parenchyma) and caused complete loss of its BBB‐penetration ability.

Thus, 2‐[^18^F]INH‐PET would be a desirable new technique that may allow for specific diagnosis of *M. tuberculosis* manifestations in most physiological compartments; however, clinical trials are the only setting to assess the true potential. That being said, based on preclinical findings, the imaging of disseminated infection in areas such as the heart, kidneys, liver and brain will most likely be difficult, due to substantial background noise and insufficient clearance within the permitting radionuclide half‐life.^[47]^ The relatively low background signal for 2‐[^18^F]INH‐PET observed in the abdominal regions would (in theory) enable a sensitive detection of *M. tuberculosis* in these regions. Thus far, no attempt has been made to analyze [^11^C]INH distribution in the presence of infection.

### 2.4 Isoniazid Analogues – Inhibitors of Enoyl‐Acyl Carrier Protein Reductase

PT70 and PT119 were developed with the MoA hinging on directly inhibiting the pathway's key enzyme, called enoyl‐acyl carrier protein reductase (InhA), without prior activation.[^52^, ^53^] Thus, the potent inhibition (*K*_i_=7.8 nM) of *M. tuberculosis* InhA by PT70 was demonstrated in vitro through the formation of a ternary PT70‐InhA‐NAD adduct that has a relatively slow dissociation (*t*_1/2_=17 minutes).^[53]^ PT119, as the cyano‐derivative of PT70, displays preferential activity (*K*_i_=0.001 nM) against *S. aureus* FabI, an InhA homologue, with a residence time of the PT119‐FabI‐NAD complex reaching 750 minutes.^[52]^ The strong target binding and long target residence time, in combination with no prior activation requirements, made these analogues of INH attractive candidates for infection‐specific radiotracer development.[^54^, ^55^]

#### 2.4.1 [^11^C]PT70

In 2015, Wang et al.^[54]^ radiosynthesized [^11^C]PT70 and measured the in situ biodistribution in healthy and methicillin‐resistant *S. aureus* (MRSA)‐infected mice, as well as healthy non‐human primates. The radiosynthesis of [^11^C]PT70 was achieved with a RCY of 40–50 % within 50 minutes by the introduction of Carbon‐11 to a tributylstannyl‐modified phenoxyphenol intermediate using a modified Stille reaction with [^11^C]CH_3_I.^[56]^ The resulting product purity was >98 %, with superior specific activities ranging from 259–481 GBq μmol^−1^. There were no significant differences in [^11^C]PT70 accumulation between healthy and infected murine muscular tissues (a plausible result as MRSA does not possess any InhA). Future studies that would use relevant infection models, such as *M. tuberculosis*‐infected mice, are recommended to evaluate the true infection‐imaging potential of [^11^C]PT70.^[54]^

#### 2.4.2 [^11^C]PT119

Wang et al. reported the radiosynthesis of [^11^C]PT119 and measured its biodistribution in situ in MRSA‐infected and healthy mice. Radiosynthesis of [^11^C]PT119 was achieved with a RCY of 30–50 % within 50 minutes by isotope introduction to an iodo‐modified phenoxyphenol intermediate by one‐step tetrakis(triphenylphosphine)palladium(0)‐catalyzed cyanation with [^11^C]HCN. The resulting product disclosed 98 % RCP with excellent specific activities ranging from 18.5–29.6 GBq μmol^−1^.^[55]^ Like [^11^C]PT70, no significant difference in [^11^C]PT119 accumulation was found between healthy and MRSA‐infected mice. Unlike [^11^C]PT70, no accumulation of [^11^C]PT119 was recorded in the presence of a relevant infection strain that harbors the *S. aureus* FabI.^[52]^

#### 2.4.3 Relevance of Radiolabeled PT70/PT119

The reason for the lack in differential accumulation of [^11^C]PT119 in infected tissue and the potential of [^11^C]PT70 are still unknown. It has been hypothesized that this might be due to the metabolic deactivation of PT70/PT119 through O‐glucuronidation, where the metabolite still houses the radioactive carbon.[^54^, ^55^] Thus, at micro‐dose levels, the radioactive signal may, in part, originate from the metabolite, which might have a reduced binding affinity to InhA/FabI. This might also explain the inability of PT70 to reduce the bacterial burden within the *M. tuberculosis*‐infected lungs in these mice. However, PET‐guided biodistribution revealed a significant signal within mice with healthy lungs. This is substantiated by PT70, which reduced the bacterial count within the spleen,^[57]^ while subsequent PET imaging revealed that pre‐dosing with PT70 more than doubled the concentration within this organ. At the same time, no such changes were recorded within the lungs.^[54]^

On the other hand, it may simply be that these tracers do not accumulate sufficiently at the site of infection to stand out from the background noise in the short imaging window permitted by carbon‐11. Therefore, delayed imaging could allow for sufficient background clearance; but to date, the reporting of quality images is limited. However, structure–activity relationship (SAR) studies revealed several time‐dependent β‐ring modified diaryl ether InhA inhibitors that contain fluorine, which could guide the development of an ^18^F‐analogue with a specific affinity to bacteria.^[57]^ Nonetheless, for now, an investigation is warranted to conclude studies on the infection imaging potential of InhA/FabI inhibitors exploiting a correct approach to the study.

### 2.5 Pyrazinamide

Pyrazinamide (PZA) is a narrow‐spectrum antibiotic exclusively active against *M. tuberculosis*. PZA plays a well‐established role as part of first‐ and second‐line treatment regimens for TB‐ and multidrug‐resistant (MDR)–TB positive patients.^[58]^ Despite PZA's efficacy in reducing mycobacterial burdens in synergy with other drugs, its mechanism of action is still unclear due to the lack of identification of definitive biological targets.^[59]^ Thus far, PZA is considered a prodrug that becomes activated by the mycobacterial amidase, pyrazinamidase (PZAase), to form pyrazinoic acid (POA). This is evident from clinical isolates resistant to PZA, where the loss of function mutations in the *pncA* gene (encoding PZAase) is the most prominent mechanism of emerging resistance.^[60]^

#### 2.5.1 [^11^C]Pyrazinamide ([^11^C]PZA)

Successful radiosynthesis for [^11^C]PZA was reported in 2010 by Liu et al.,^[48]^ involving the initial production of a [^11^C]cyanopyrazine intermediate from 2‐iodopyrazine and [^11^C]HCN, followed by hydrolysis of the cyano group with hydrogen peroxide, under basic conditions for five minutes. Radiochemically‐pure [^11^C]PZA (>99 %), with a specific activity >4.4 GBq μmol^−1^, was produced with a decay‐corrected yield (calculated from [^11^C]HCN) above 50 % in 45 minutes. The in vivo biodistribution in healthy baboons, using [^11^C]PZA‐PET, revealed that [^11^C]PZA and/or its metabolites rapidly cleared from the blood pool to the major organ compartments, including the myocardium, liver, lungs and kidneys; but rapidly washed out from all relevant tissues (except for the kidney medulla and bladder). Interestingly, [^11^C]PZA penetrated the brain tissue with exceptional efficiency. Thus far, [^11^C]PZA has not been further evaluated as an infection imaging agent.

#### 2.5.2 5‐[^18^F]F‐Pyrazinamide (5‐[^18^F]F‐PZA)

Zhang et al. successfully synthesized 5‐[^18^F]F‐PZA and evaluated its potential as a mycobacterial‐specific radiotracer in a pulmonary *M. tuberculosis*‐infected mouse model.^[61]^ 5‐[^18^F]F‐PZA was synthesized from 5‐chloro‐PZA through halogen exchange of the chlorine with [^18^F]fluoride. An overall decay‐corrected yield of 25 % and specific activity above 2.5 GBq μmol^−1^ were achieved within 60 minutes. No selective in vitro accumulation was found in *M. tuberculosis* compared to *E. coli*, *S. aureus*, *P. aeruginosa*, or J774A‐1 murine macrophage cells. Unfortunately, modification of PZA to 5‐[^18^F]F‐PZA resulted in decreased recognition by the PZAase enzyme and subsequent loss of prodrug activation to 5‐[^18^F]F‐POA. This result is substantiated by a more than 100‐times higher MIC against *M. tuberculosis* compared to unmodified PZA.^[61]^ As predicted, 5‐[^18^F]F‐PZA‐PET imaging of pulmonary TB‐infected mice indicated no significant difference in the accumulation of 5‐[^18^F]F‐PZA between uninfected and infected lung tissues.^[61]^ Another concern noted by the authors was the high uptake within bone tissue. Cold 5‐F‐PZA was found to be rapidly metabolized by CD‐1 mouse liver homogenates, and defluorination was detected as early as two minutes after incubation, which is an indication that 5‐F‐PZA is unstable in vivo.

#### 2.5.3 Relevance and Limitations of Radiolabeled Pyrazinamide

Regarding their biodistribution, both structurally conserved [^11^C]PZA and modified 5‐[^18^F]F‐PZA elicit similar properties favorable for infection‐imaging applications, of which its excellent brain penetration is a sought‐after trait. Unfortunately, PZA's activation and anti‐mycobacterial activity is highly conserved within its structure, which is evident from the deficient uptake and specificity of 5‐[^18^F]F‐PZA by *M. tuberculosis* cell cultures. Although 5‐[^18^F]F‐PZA's clinical applicability as a mycobacterial specific radiotracer has been dismissed, recent evidence points to a retention of treatment efficacy when POA is administered intravenously.[^62^, ^63^] Future PZA‐based radiotracer designs may be simplified by radiolabeling POA to bypass complicated host‐mediated/mycobacterial activation processes.

### 2.6 Linezolid

Linezolid is a broad‐spectrum antibiotic reserved for treating drug‐resistant Gram‐positive and only selected Gram‐negative bacterial infections.^[64]^ The United States Food and Drug Administration (FDA) has approved linezolid for the treatment of MDR‐TB and extensively drug‐resistant (XDR)‐TB as part of a three‐drug treatment combination, which includes bedaquiline and pretomanid.^[65]^ As part of the oxazolidinone antibiotic class, linezolid selectively inhibits the synthesis of bacterial proteins by directly binding to the bacterial 23S ribosomal RNA of the 50S subunit, essentially blocking the formation of a functional 70S ribosomal initiation complex and the subsequent initiation of the RNA translational process.^[66]^ The bacterial selectivity of linezolid over *Mammalia* is attributed to the substantial difference in prokaryotic and eukaryotic ribosomal structure and function.

#### 2.6.1 [^18^F]F‐Linezolid

Mota et al. recently radiosynthesized and utilized [^18^F]F‐linezolid as a radiotracer to study its in situ pharmacokinetics within a pneumonic‐TB mouse model.^[67]^ [^18^F]F‐linezolid was synthesized through copper‐mediated radio‐fluorination of a boronic ester precursor derivative of linezolid. The radiochemical yield ranged between only 1 and 3 %, with a radiochemical conversion between 2 and 15 %; but it was >95 % radiochemically‐pure (no specific activity was provided). Following intravenous tracer administration, the PET image‐derived pharmacokinetic data demonstrated rapid distribution of [^18^F]F‐linezolid to all major organs and subsequent hepatobiliary and renal elimination.^[68]^ Furthermore, the authors also showed how [^18^F]F‐linezolid efficiently penetrated TB‐infected lung foci, as previously demonstrated.^[69]^ Unfortunately, [^18^F]F‐linezolid penetrated both infected and healthy lung tissue without discrepancy; and in both cases, high signals were achieved with AUC(_tissue/plasma)_ ratios reaching above 1.0. This was in line with the insignificant accumulation of [^18^F]F‐linezolid found in viable mycobacterial cultures over its heat‐killed control culture.

#### 2.6.2 Relevance and Limitations of Radiolabeled Linezolid

The insignificant uptake of [^18^F]F‐linezolid within mycobacterial cultures in both in vivo and in vitro experimental settings discourages use of this tracer for infection imaging. To this end, the lack in tracer uptake is puzzling since no structural alterations were made in the radiolabeling process of this antibiotic; thus, target binding affinity was expected to be maintained. It may be warranted to further investigate the underlying cause, whether it be radiotracer formulation‐ or antibiotic MoA‐related. In addition, linezolid is active against those Gram‐positive bacteria causing resistant disease (vancomycin‐resistant enterococci or MRSA); hence [^18^F]F‐linezolid could be investigated beyond imaging of TB.

## 3 Radio‐Antibiotic PET Imaging for Pharmacologic Drug Characterization

Plasma pharmacokinetic (PK) measurements are considered essential within the developmental pipeline for every novel antibiotic and for determining optimal drug dosing regimens. However, it is known that plasma PK measurements do not always correlate well with intralesional PK, which is dependent on both drug characteristics and host factors, such as lesion pathology, that may restrict or alter drug bioavailability.^[70]^ The direct measuring of drug bioavailability within these tissues with traditional quantitative tools that rely on invasive procedures such as tissue resection remains a challenge. Traditional quantitative tools are also limited to a single time‐point measurement of a sample gathered from a single lesion. Lesion pathology often displays inter‐ and intra‐patient heterogeneity that evolves over time. Thus, a single lesion/time‐point measurement in itself introduces sample bias.[^71^, ^72^] In this light, as part of the next section, we will highlight the non‐invasive use and potential of PET in supporting imaging‐derived PK/PD studies using radiolabeled antibiotics.

### 3.1 Erythromycin

Erythromycin is a broad‐spectrum antibiotic prescribed to treat a wide variety of Gram‐positive and Gram‐negative bacterial infections. Being of the macrolide antibiotic class, erythromycin exerts a bacteriostatic effect by selectively inhibiting the synthesis of bacterial proteins. The antibiotic's MoA involves the disruption of 70S ribosomal complex assembly through direct binding to the 23S ribosomal RNA section located on the 50S ribosomal sub‐unit, essentially disrupting the bacterial RNA translation process.^[73]^ In 1982, Pike et al. had already successfully radiolabeled erythromycin with carbon‐11.^[74]^ Within the same year, Wollmer et al. utilized [^11^C]erythromycin as a radiotracer in a novel technique that utilized the quantitative capabilities unique to PET to study erythromycin's in situ pharmacokinetics in a pneumonic mouse model.^[75]^

#### 3.1.1 [^11^C]Erythromycin

[^11^C]erythromycin synthesis involves the reductive methylation of *N*,*N*‐dimethylerythromycin A with [^11^C]formldehyde.^[74]^ Useful activity amounts (56–185 MBq) were provided with a decay‐corrected yield of 4–12 % (calculated from [^11^C]CO_2_) in 42 minutes. The PET image‐guided pharmacokinetic results showed rapid penetration of [^11^C]erythromycin into healthy and pneumonic lung tissue. Yet, no significant differences were observed in radiotracer concentration between these two compartments over 45 minutes.^[75]^ To date, no subsequent studies have been conducted to further evaluate the potential of [^11^C]erythromycin for nuclear imaging.

### 3.2 Rifampin

Rifampin is an antibiotic prescribed for the treatment of Gram‐positive bacterial infections, predominantly against TB; and selected Gram‐negative bacterial infections.^[76]^ This drug binds directly to bacterial RNA polymerase and forms a relatively stable inactive enzyme–drug complex (disassociation constant, *K*_d_ ≤3.0 nM, *t*_1/2_≥9 minutes). This interaction essentially blocks bacterial DNA transcription and ultimately results in cell death.^[77]^ Even though both Gram‐positive and ‐negative bacteria display the same rifampin–RNA polymerase binding affinity, the latter is significantly less susceptible to rifampin exposure (MIC ≈0.01 μg ml^−1^ and 8–32 μg ml^−1^, respectively) due to the limited penetration of the drug through their physiologically distinct cell walls.^[78]^ In contrast, RNA polymerases of eukaryotic origin are unresponsive to rifampin exposure, with no inhibition occurring at concentrations up to ×10^4^ higher than the effective dose for bacteria (ED_50_, ≈0.01 μg ml^−1^).^[79]^

#### 3.2.1 [^11^C]Rifampin

In 2010, Liu et al. reported the first successful radiosynthesis of [^11^C]rifampin ([^11^C]RIF) and used PET imaging to study its pharmacokinetic parameters in healthy non‐human primates.^[48]^ Later, a series of subsequent reports described in detail the spatial–temporal evolution of [^11^C]RIF exposure to diverse TB lesions manifested within a panel of small animal models and human patients.[^71^, ^80^, ^81^, ^82^, ^83^] The piperazine moiety of rifampin was radiolabeled with [^11^C]CH_3_I within 10 minutes in a reaction solution consisting of potassium carbonate dissolved in DMSO and MeCN and heated to 110 °C. [^11^C]RIF product was subsequently purified via reverse‐phase HPLC and the acquired product fractions were concentrated in vacuo after the addition of ascorbic acid (to prevent oxidation). This method produced a radiochemically‐pure [^11^C]RIF product with excellent specific activity (>21.5 GBq μmol^−1^) in a total synthesis time of 50 minutes and with an average decay‐corrected yield greater than 15 % (calculated from [^11^C]CH_3_I).^[48]^ The literature concerning [^11^C]RIF‐PET/CT studies and biodistribution data shows that the tracer behaves similarly in healthy mice, rabbits, baboons and human volunteers in terms of blood clearance, tissue distribution, BBB penetration and elimination routes.[^71^, ^80^, ^81^] PET imaging up to 90 minutes post injection revealed that [^11^C]RIF rapidly clears from blood plasma and is distributed to all parts of the body. It reaches concentrations that are several‐fold higher than the MIC for *M. tuberculosis*, even in the brain with restricted BBB penetration (10–20 % of plasma concentration). [^11^C]RIF rapidly accumulates in the liver and mainly undergoes hepatobiliary excretion. Data from the PET/CT imaging of animals and patients with pulmonary TB undergoing rifampin‐based treatment revealed significantly decreased drug exposure and penetration into lesions compared to unaffected tissues. Furthermore, pooled PET/CT data from the 12 TB patients who participated in the study, of which six had cavity lesions, revealed spatially compartmentalized [^11^C]RIF exposure between infected lesions and cavity walls (*P*=0.023), the latter being less exposed to the drug.^[71]^ In contrast, a similar study in rabbits and a single patient with TB meningitis, also undergoing rifampin‐based treatment, revealed a statistically uniform [^11^C]RIF distribution between infected brain lesions and unaffected brain tissue.^[81]^ Due to the limited penetration of [^11^C]RIF into infectious lesions, its future use as a bacterial imaging agent seems highly unlikely; however, the pharmacokinetic radioanalysis of tissue perfusion should be encouraged.

### 3.3 Bedaquiline

Bedaquiline is a narrow‐spectrum antibiotic with selective activities towards the *Mycobacteria* genus. Its use is reserved as a last resort treatment for exclusively active pulmonary MDR‐TB and XDR‐TB infection, where it is prescribed as part of a three to four‐drug combination therapy.[^65^, ^84^] It is a diarylquinoline that specifically inhibits the proton pump of mycobacterial adenosine 5′‐triphosphate (ATP) synthase, which results in the disruption of bacterial energy production (Figure 1).^[85]^ Dormant and non‐replicating mycobacteria have also been found to be susceptible to it, due to energy metabolism still being required to maintain this state, albeit significantly less than normal. Despite ATP synthase being a highly conserved enzyme between eukaryotes and prokaryotes, bedaquiline was found to be up to 20 000‐fold less potent at inhibiting human mitochondrial ATP synthase compared to that of mycobacterial ATP synthase (IC_50_ <10 nM).^[86]^

#### 3.3.1 [^76^Br]Br‐Bedaquiline

In 2019, Ordonez et al. reported on the radiolabeling of bedaquiline with bromine‐76 ([^76^Br]Br‐BDQ) and PET imaging of the in situ PK/PD drug profile using a murine TB model.^[87]^ Radiosynthesis of [^76^Br]Br‐BDQ involved the initial conversion of the bromine group to a boronic ester intermediate with bis(pinacolato)di‐boron and a

[1,1′‐bis(diphenylphosphino)ferrocene]palladium(II) dichloride complex. This boronic ester was subsequently converted to [^76^Br]Br‐BDQ using NH_4_^76^Br in the presence of a copper catalyst. A radiochemical yield of 6 % (non‐decay corrected), after preparative HPLC purification, was achieved for which the specific activity was not reported. PET/CT image quantification of intralesional drug penetration in conjunction with 2D autoradiography and tissue staining revealed that [^76^Br]Br‐BDQ effectively penetrates infectious lung lesions with AUC_(tissue/plasma)_ ratios reaching above 0.85. However, radiotracer penetration was spatially heterogeneous and significantly reduced within infective lesions, and even less penetration was observed into caseous TB granulomas. The general biodistribution of [^76^Br]Br‐BDQ concerned all major organs, particularly hepatic tracer accumulation and with a high presence in adipose tissue. Limited [^76^Br]Br‐BDQ penetration into the brain parenchyma was noted. The limited lesion penetration and unfavorable pharmacokinetics discourage using [^76^Br]Br‐BDQ as a potential TB‐specific radiotracer.

### 3.4 Clinical Relevance of Antibiotic Radio‐Isotopologues for Pharmacologic Drug Characterization

From the emerging PET PK/PD studies presented in the above review section, it is clear that PET is a clinically translatable tool for non‐invasive and longitudinal measurement of intralesional antimicrobial drug distribution in infected tissues. For instance, from the series of PK/PD studies using [^11^C]RIF, the multi‐compartmental (lungs, brain and bone) quantification of spatially heterogeneous and highly variable tracer exposure to multiple distinct lesions within an individual patient was possible. So far, and considered as uncharted territory, the image‐guided analysis revealed the presence of multiple, but variable, pathophysiological responses unique to each lesion that can affect drug bioavailability.[^71^, ^81^, ^82^, ^83^] It is known that inappropriate levels of antibiotic bioavailability and pathogen exposure can lead to treatment failure and the selection of resistant organisms. Thus, more detailed pharmacologic drug characterization may have significant implications in future treatment optimization efforts.[^71^, ^88^, ^89^] PET imaging may hereby aid in elegantly translating results from pharmacokinetic modelling on novel antibiotics. Additionally, by radiolabeling potential antibiotic candidates, the acquisition of longitudinal multi‐compartment pharmacological data is feasible (non‐invasively and at sub‐therapeutic doses to avoid toxicity). As an example, PET biodistribution of the radiolabeled experimental drug, [^11^C]PT70, revealed that pre‐dosing with PT70 more than doubled drug concentration within the spleen, which may explain the increased drug efficacy, specifically in this region. Longitudinal profiling in the same subjects at several points in time can be performed, thereby reducing animal‐to‐animal variability, as well as the costs associated with sacrificing different animal cohorts at each time‐point. Finally, because PET is very much translatable into the clinical setting, it will also allow for early proof‐of‐concept studies that typically require 20 patients and are highly encouraged by the FDA.[^71^, ^72^]

## 4 Critical Analysis

### 4.1 Summary and Validation

The superior parameters of PET (sensitivity/resolution), its advanced technology (real‐time, whole‐body imaging/quantification), and the global impact of a growing PET infrastructure offer opportunities to develop new strategies that could revolutionize the management of patients with infectious diseases.^[90]^ There are 20 antibiotic‐derived PET radiotracers with a bacterial‐specific binding mechanism, or MoA, reported in the literature (Table 1). These PET radiotracers have been derived from 11 FDA‐approved antibiotics that can be classified into three categories: broad‐spectrum antibiotics, narrow‐spectrum mycobacterium tuberculosis antibiotics, and a Gram‐positive selective antibiotic (linezolid). From the radiochemical viewpoint, these PET radiotracers can be divided into two groups: structurally modified antibiotic radiotracers and antibiotic radio‐isotopologues (structurally unaltered antibiotic radiotracers).

**Table 1:** Overview of antibiotic‐derived PET radiotracers.^**[**a–c**]**^

Radiotracer	Specific activity [GBq μmol^−1^]^[d]^	State (D/P/C)^[e]^	SBU (Y/N)^[f]^	Application^[g]^	Ref.
[^11^C]trimethoprim 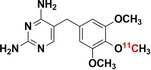	19–37	C	Y	IOI	[20]
[^18^F]F‐propyl‐trimethoprim 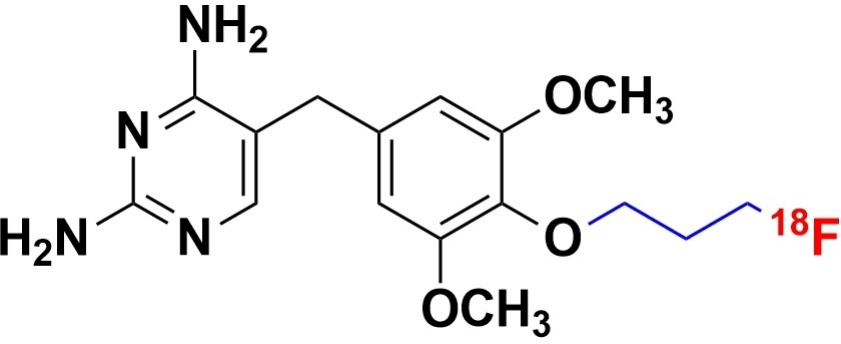	185–555	C	Y	IOI	[18]
[^18^F]F‐ciprofloxacin 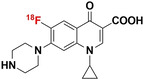	0.43±0.20	C	N	IOI	[32, 33, 34]
[^68^Ga]Ga‐*p*‐SCN−Bz‐NOTA‐ciprofloxacin (1) 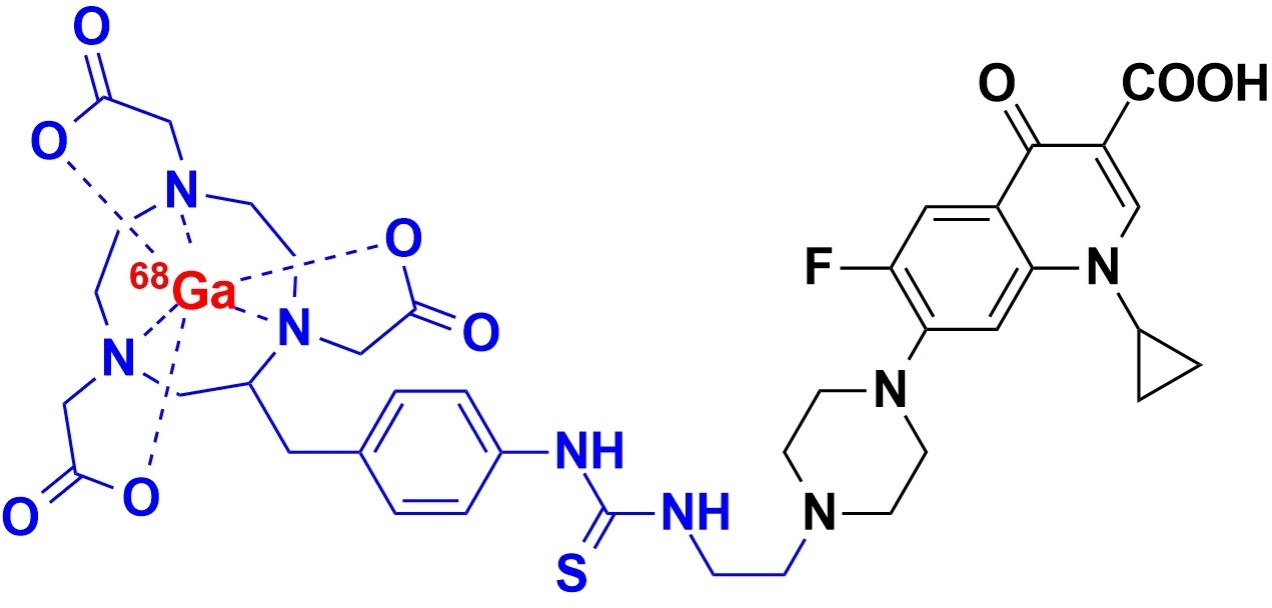	6.2±0.4	P	Y	IOI	[28]
[^68^Ga]Ga‐*p*‐SCN−Bz‐DOTA‐ciprofloxacin (2) 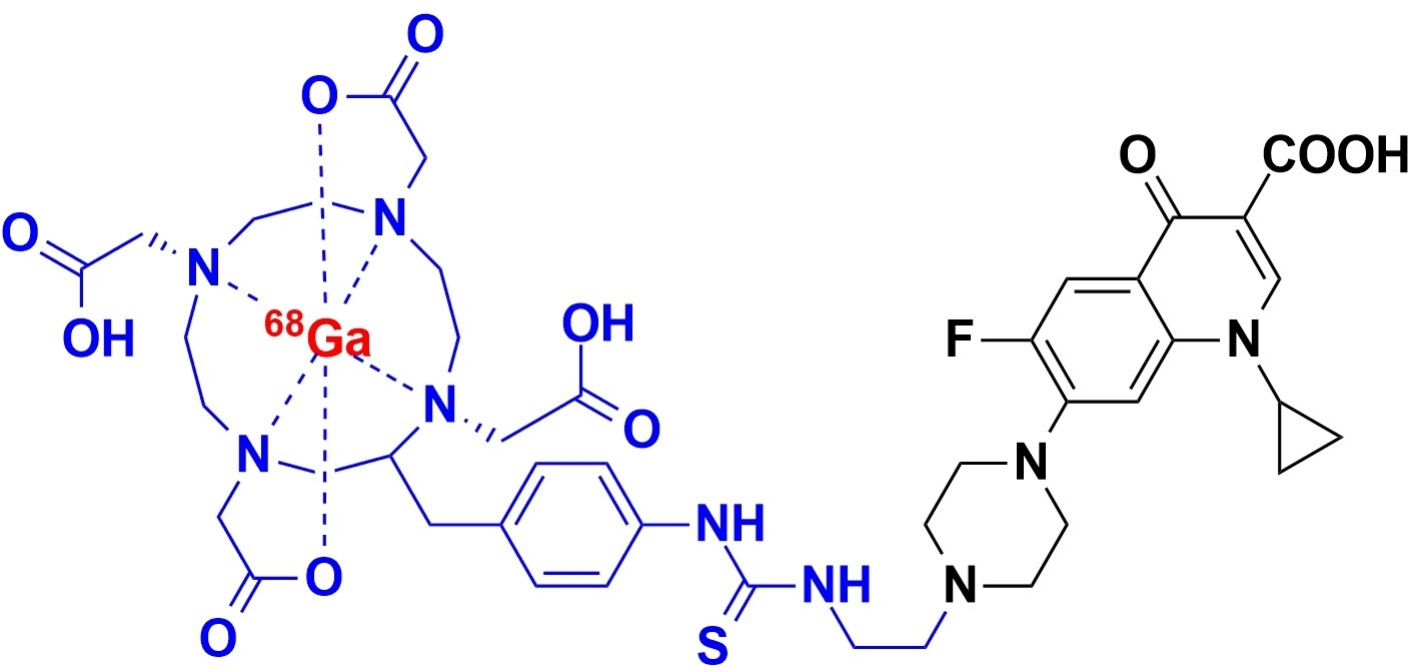	6.2±0.4	P	Y	IOI	[28]
[^68^Ga]Ga−DOTA‐ciprofloxacin 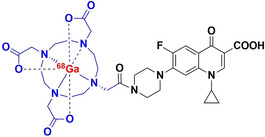	nd	D	Y^**[i]**^	IOI	[35]
N4′‐3‐[^18^F]F‐propyl‐ciprofloxacin 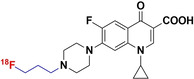	149±75	D	Y	IOI	[27]
[^18^F]F‐lomefloxacin 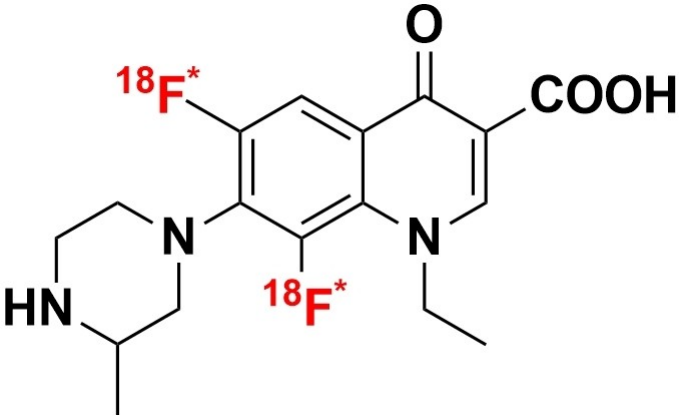	nd	C	nd	PK/PD	[31]
[^18^F]F‐fleroxacin 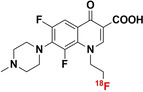	1.85	C	nd	PK/PD^[h]^	[29, 36, 37, 38]
[^18^F]F‐trovafloxacin 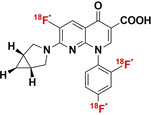	nd	C	nd	PK/PD^[h]^	[30, 39, 40]
[^11^C]isoniazid 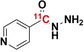	5.0	P	nd	PK/PD	[48]
2‐[^18^F]F‐isoniazid 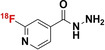	0.007–0.011	P	Y	IOI	[45, 46, 47]
[^11^C]PT70 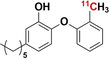	259–481	P	nd	PK/PD^[h]^	[54]
[^11^C]PT119 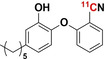	19–30	P	nd	PK/PD^[h]^	[55]
[^11^C]pyrazinamide 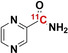	4.4	P	nd	PK/PD	[48]
5‐[^18^F]F‐pyrazinamide 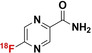	2.5	P	N	IOI	[61]
[^18^F]F‐linezolid 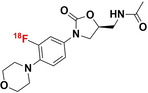	nd	P	N	IOI/PK/PD^[h]^	[67]
[^76^Br]Br‐bedaquiline 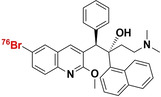	nd	P	nd	PK/PD^[h]^	[87]
[^11^C]rifampin 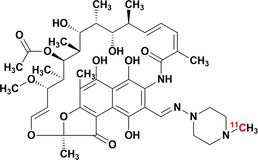	278	C	nd	PK/PD^[h]^	[48, 71, 80, 81, 82, 83]
[^11^C]erythromycin 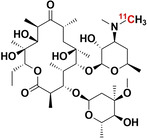	nd	C	nd	PK/PD^[h]^	[75]

[a] Blue bonds indicate structural modification to core antibiotic structure; [b] red atoms indicate radioisotope incorporation; [c] ^18^F* indicates that the position of ^18^F was not determined; [d] nd indicates this value was not determined from the literature; [e] development/preclinical/clinical; [f] specific bacterial uptake (in vitro), yes or no; [g] purpose of application, either imaging of infection (IOI), or pharmacokinetic and pharmacodynamic (PK/PD) measurement; [h] PK/PD measurement in the presence of infection (in situ); [i] conclusion made on preliminary results.

#### 4.1.1 Structurally Modified Antibiotics

The structurally modified antibiotics were specifically designed and tested for imaging infections. The structural modifications (Table 1, colored blue) required to incorporate PET‐radionuclides for radio‐fluorination (2‐[^18^F]INH and 5‐[^18^F]F‐PZA); the introduction of a fluoropropyl spacer moiety ([^18^F]FP‐TMP and [^18^F]16); chelator functionalization (DOTA/NOTA‐ciprofloxacin, e.g., [^68^Ga]Ga‐1/‐2 and [^68^Ga]Ga‐DOTA‐ciprofloxacin); and direct radiolabeling of experimental antibiotic analogues ([^11^C]PT70 and [^11^C]PT119). To date, seven of these nine radiotracers have been tested in preclinical animal models to evaluate their bacterial‐specific imaging potential. Even though [^18^F]FP‐TMP, 2‐[^18^F]INH, and [^68^Ga]‐1/2 are capable of distinguishing bacterial infection from inflammatory processes, both in vitro and in vivo, only [^18^F]FP‐TMP has reached clinical trials. [^11^C]PT70 also underwent similar in vivo evaluation, however, the infection was induced with an irrelevant bacterial strain. [^18^F]16 showed bacterial‐specific accumulation in vitro, but no in vivo studies were performed. And lastly, although in vitro bacterial uptake of [^68^Ga]Ga‐DOTA‐ciprofloxacin was tested, no firm conclusion could be drawn from this study due to lack of negative controls.

#### 4.1.2 Antibiotic Radio‐Isotopologues

Radio‐isotopologues were mainly developed and used to study in vivo PK/PD parameters. Only [^11^C]TMP, [^18^F]F‐ciprofloxacin and [^18^F]F‐linezolid have been evaluated specifically for both in vitro and in vivo bacterial uptake. Only five of eleven radiotracers were used to specially study PK/PD properties in infected animals and healthy/infected patients. These include [^18^F]F‐fleroxacin, [^18^F]F‐trovafloxacin and [^76^Br]Br‐BDQ, which were exclusively done in animals, while [^11^C]RIF and [^11^C]erythromycin were also examined in humans. In addition, [^11^C]INH, [^11^C]PZA and [^18^F]F‐lomefloxacin were used only for PK data purposes in healthy non‐human subjects. [^11^C]TMP was identified as a superior infection‐imaging agent because it distinguished bacterial infection from inflammatory disorders with a high level of success in the patient case studies presented. In addition, [^11^C]TMP showed promising results in accurately monitoring disease pathogenesis and response to antibiotic treatment in two patients: one with CF presenting TMP resistant *E. coli*‐positive sputum samples; and one with biopsy‐proven MSSA discitis.^[20]^ Conversely, [^18^F]F‐ciprofloxacin and [^18^F]F‐linezolid performed poorly as infection‐specific imaging agents due to a lack of bacterial uptake. For the remaining radiotracers, little bacterial imaging potential can be extrapolated from these PK studies due to limitations in experimental design. The most notable limitation is that most subjects had already undergone pre‐dosing with corresponding unlabeled antibiotics.

#### 4.1.3 “Unclear” Imaging of Infection and the Lack of Translational Follow‐Through

As outlined above, antibiotic‐derived PET radiotracer development was, and remains, very limited and fragmented into single, small studies, often with incoherent study designs; and hence does not allow for firm conclusions to be drawn. It is argued that the most promising radiotracer candidates remain confined to the pre‐clinical level, due to a lack of standardized protocols, which do not introduce these biases. In turn, this reduces the validity and reliability of otherwise promising results, thus hampering clinical translation efforts due to a lack of confidence and a solid research strategy to build upon.[^13^, ^91^] The latter has been exemplified in a recent systematic review by Auletta et al., which included all infection‐specific PET radiotracers, with an in vivo evaluation of bacteria published between 2005–2018.^[91]^ Out of 35 studies, only 11 studies were identified as having low‐risk bias. The main sources of bias were related to animal model selection and the origin of bacterial cells as well as issues related to controls and experimental settings: bacterial burden; radiopharmaceutical dose and specific activity; administration route; imaging time frames; and time intervals between bacteria injections. It is noteworthy that a possible bacterial mutation after inoculation was not considered in any of the published studies. In this regard, [^11^C]TMP and [^18^F]FP‐TMP show promise that antibiotics can be clinically relevant infection‐imaging agents due to the high quality and extensive study designs employed. These include: excellent radiotracer‐specific activity; enzymatic and whole‐cell in vitro characterization of specific uptake; the use of multiple negative controls and antibiotic‐treated bacteria; testing uptake in a broad panel of bacteria that includes various resistance mechanisms and gene‐knockouts; genetic characterization of bacteria tested, in both in vitro and in vivo settings; using relevant in vivo animal models that fully capitulate infection pathology in humans, including sterile inflammation and cancer; characterization of normal biodistribution and pharmacokinetic parameters; preliminary imaging with known and well‐characterized infection/disease pathology; analysis of tracer performance compared to standard tracers used in clinical setting ([^18^F]FDG); and assessing tracer performance in preliminary case studies. With this newly‐found confidence, further evaluation of [^11^C]INH, 2‐[^18^F]INH, [^18^F]16, and [^68^Ga]Ga‐1/‐2 can be expected soon.

### 4.2 Going forward?

There is currently no robust research platform dedicated to the imaging of infections that could facilitate streamlined development and evaluation of novel infection‐specific radiotracers.^[13]^ To overcome the current drawbacks, a focus on using evidence‐guided radiotracer design strategies to cater for individually desired applications/needs is required. This would include, but is not limited to, choosing between broad‐range or pathogen‐specific imaging; targeting intra‐ or extracellular pathogens; monitoring response to treatment; or monitoring the emergence of bacterial antibiotic resistance mechanisms. This strategy may be more efficient in creating a solid infection‐specific PET platform with available and reliable radiopharmaceutical tools to address clinical needs; but also, to study infectious diseases from different angles and profiting more from the data provided from (radio)kinetic analysis ‐ similar to the approach shown to be successful in advancing the imaging of neuropharmacology.

The development of such a platform dedicated to the PET imaging of infectious diseases has been slow, which can be attributed to several reasons. Over the years, potential root causes have been continually reported[^4^, ^8^, ^13^, ^14^, ^72^, ^91^, ^92^] and may be categorized as follows: i) difficulties with adapting strategies for antibiotic vector selection; ii) challenges in preclinical study design; iii) clinical translation barriers; and iv) lack of adequate research funding, not reflecting the global morbidity and mortality caused by bacterial infections.

As mentioned before, unclear study designs often resulted in skepticism regarding whether candidate infection radiopharmaceutical performances were fully explored.^[91]^ Thus, there is an unmet need to implement standardized protocols and to develop consensus guidelines/recommendations on animal infection models, preferably written by a joint technical committee.^[13]^ Fortunately, there is increased awareness of study design flaws, but we are still far from addressing these issues with a coherent/standardized approach. In this regard, key considerations in the development and clinical translation of bacteria‐specific imaging agents, in general, have been thoroughly and comprehensively reviewed.[^4^, ^13^, ^72^] Here, we will highlight the important aspects uniquely applicable to antibiotic‐based radiotracers (Table 2).

**Table 2:** Challenges and possible solutions for the development and testing of novel antibiotic‐based radiopharmaceuticals for infection imaging.

Challenge		Possible strategy/solution		Limitation
Antibiotic radiolabeling protocol is unable to match its antibiotic MoA		‐ libraries & SAR (target binding efficacy) ‐ computational tests (aim at preserving the pharmacophore)		‐ radioisotope production and radiopharmaceutical aspects (low specific activity)
Risk of compromised tracer sensitivity		‐ select antibiotics that target highly active/expressed biological processes ‐ disregard antibiotics with MoA that are not well understood ‐ consider the mass effect of tracer formulation ‐ radiosynthesis optimization (formulation, dosage, carrier content); following quality guidelines ‐ testing tracer sensitivity in non‐human primate models or first‐in human investigations prior to clinical trials		‐ biological target expression is underwhelming ‐ threshold *B*_max_^[a]^/*K*_d_^[b]^ may decrease <10 for antibiotics derivatives ‐ radiotracer: inadequate specific activity ‐ small animal models only acceptable for proof‐of‐principle investigation
Risk associated with accuracy of visualizing infection		‐ disregard antibiotics with predisposed MoA ‐ drug resistant pathogens: use vectors that circumvent/take advantage of defense mechanism, i.e., target overexpression or genetic redundancy		‐ presence of additional (cold) ligand or conflicting pathogen environment ‐ cumbersome prodrug activation processes ‐ pre‐treated subjects using widely prescribed antibiotics
Effects of empiric use of antibiotics		‐ opting for radiosynthesis of antibiotics with unique MoA is crucial		‐ radiotracer: inadequate specific activity
Unwanted (altered) tracer bioavailability and biodistribution		‐ ADME: prioritize antibiotics with rapid clearance from high‐risk organ/compartments for infection ‐ assess candidates for host enzymatic and tissue specific interactions ‐ practice SAR‐guided incorporation of a radiolabeled functional group ‐consideration of liposome‐, nanoparticle, or microsphere‐based delivery system (transfer intact tracer to target) ‐ permit radionuclide incorporation only to non‐cleavable structures		‐ relatively long biological half‐life of antibiotics ‐ antibiotics sometimes associate with host inflammatory response ‐ unforeseen shifts in physico‐chemical properties (lipophilicity by carbon chain spacers/polarity by metal chelator conjugation) can occur

[a] target protein/receptor density; [b] binding disassociation constant.

### 4.3 Required Properties for Radiotracers to Image Infections?

According to Welling et al., a clinically relevant infection tracer should ideally possess the following properties: i) high affinity and strong binding to bacteria to generate highly sensitive/specific images with high resolution; ii) capacity to penetrate mammalian cell walls to target intracellular pathogens; iii) favorable biodistribution with fast background clearance; iv) safe administration and no serious adverse events; v) high in vivo stability; and vi) easy to synthetize at relatively low cost in a good manufacturing process (GMP) set‐up.^[92]^

One of the key issues for a radiotracer remains its sensitivity and specificity to detect bacteria. Ordonez et al. recommended that it should be able to reliably detect a minimum of 10^5^ CFU/ml and achieve selective bacterial accumulation with a signal up to 100–2000‐fold above that of the host tissue.^[72]^ However, even higher sensitivity is required to detect chronic infections, which typically have a lower bacterial burden. In addition, Signore et al. argued that it should reliably be taken up by a broad range of clinically relevant pathogens, including antibiotic‐resistant strains.^[13]^ Lastly, to promote clinical translation and widespread use, it should also have utility beyond diagnosing infections, such as monitoring disease pathogenesis or response to treatment.^[90]^ Thus, a radiotracer should ideally display localized uptake proportional to its target burden, exclusively in living bacteria. So far, identifying a suitable vector candidate that fulfils most of these criteria has been very challenging.

### 4.4 Challenges and Possible Solutions for Prospective Radio‐Antibiotics

**Figure 2:**
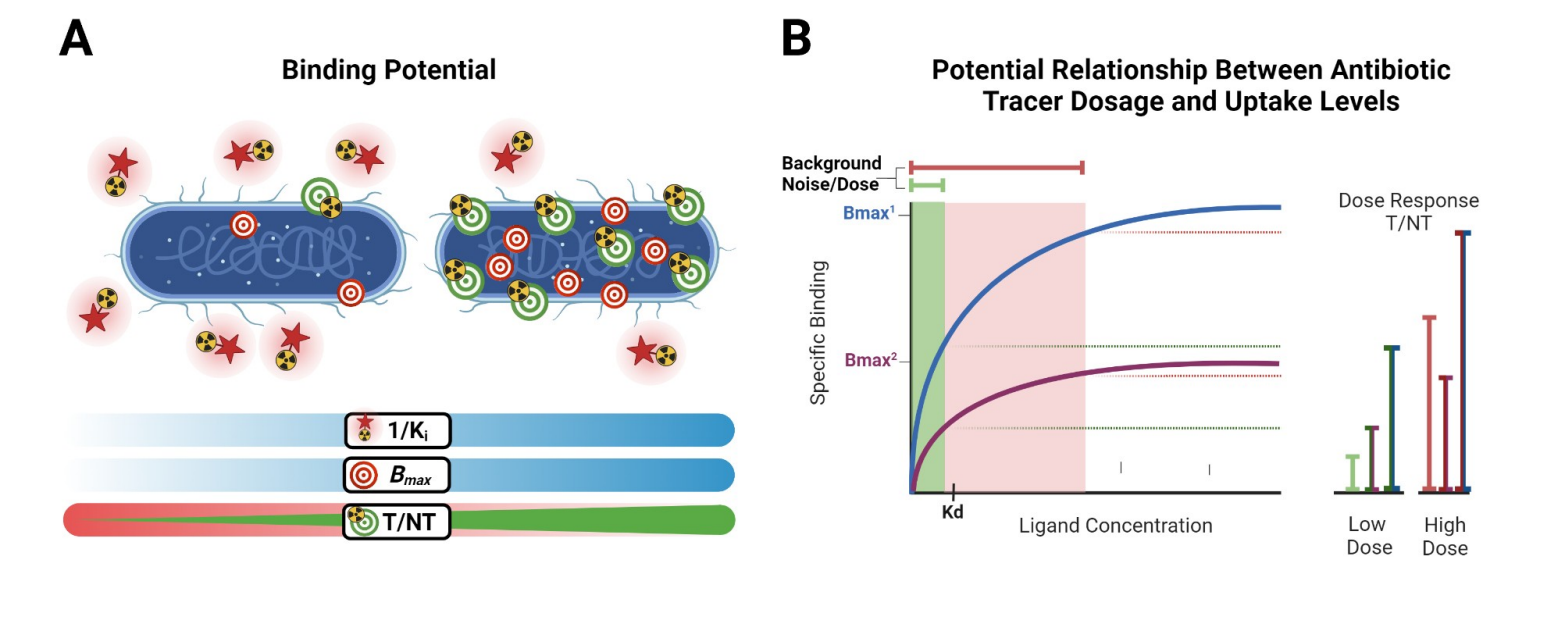
A) Illustration showing the parameters that influence the “binding potential” of a tracer to its target, a concept used in receptor imaging with radiopharmaceuticals that display a saturable binding mechanism; B) Scatchard plot showing a typical equilibration curve for a ligand binding to a saturable receptor population, and how different tracer dosages may influence specific uptake levels normalized to background noise. Additionally, this concept is used in receptor imaging where quantitative PET enables measurement of specific binding at equilibrium at various concentrations (often 6–12) of the radioligand to determine receptor number (*B*_max_) and affinity (*K*_d_) in situ. Created with Biorender.com.

**Figure 3:**
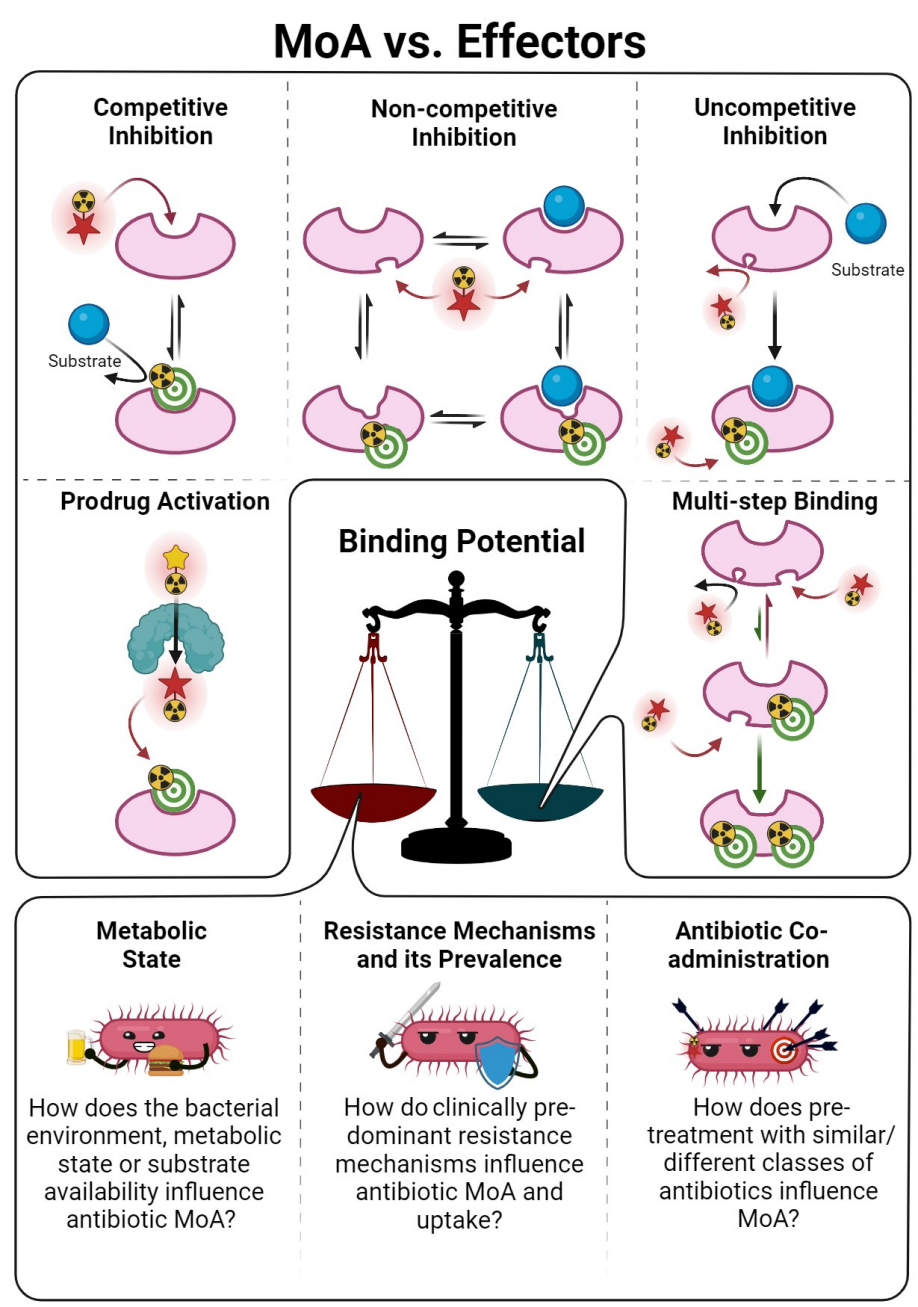
Enzyme–drug interaction characteristics of antibiotics, and their influencers, which should be taken into consideration before the development of an antibiotic radiotracer. Created with Biorender.com.

**Figure 4:**
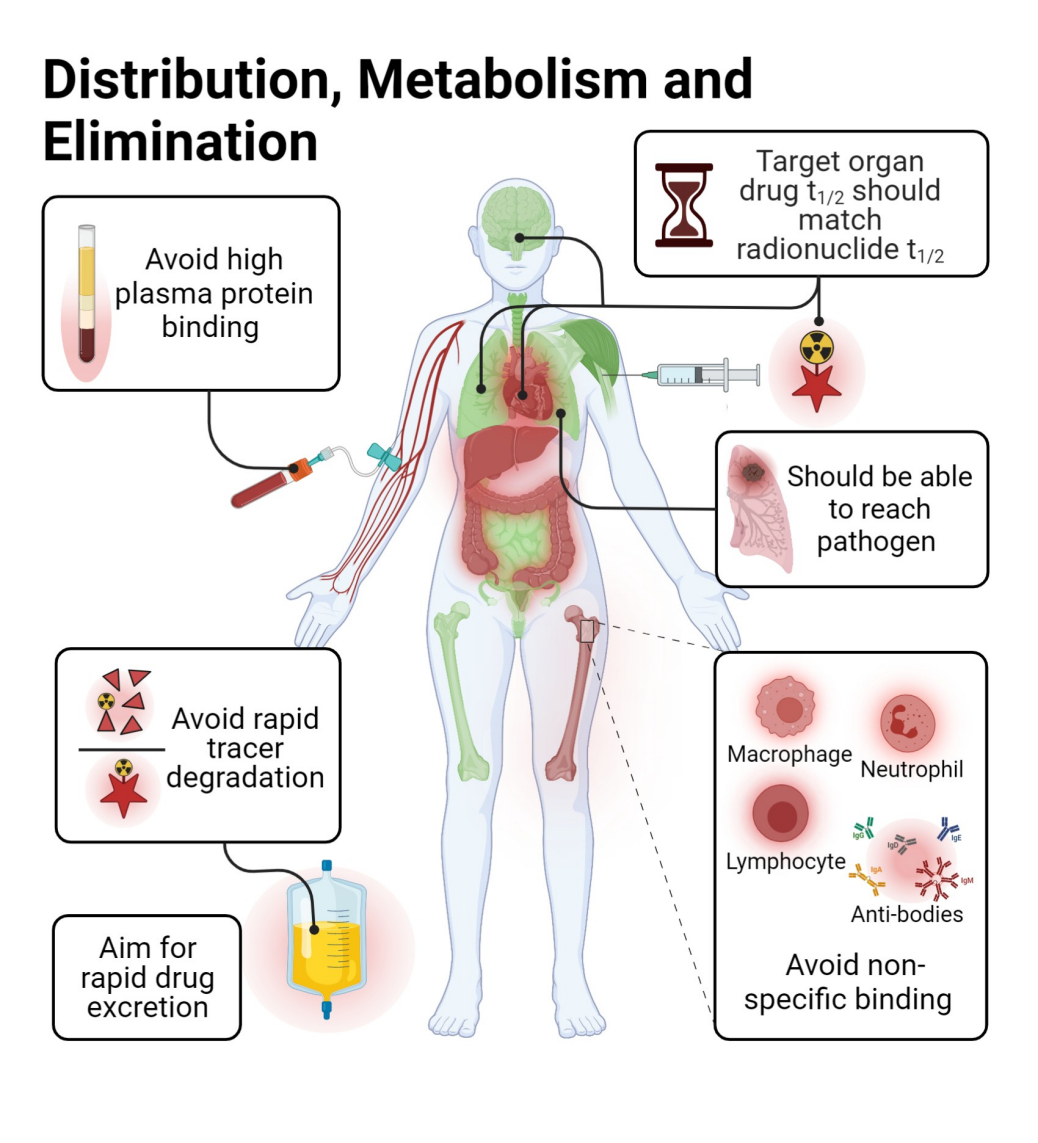
Pharmacokinetic/pharmacodynamic characteristic guidelines for selecting a prospective antibiotic for radiopharmaceutical development. Created with Biorender.com.

The vast amount of data assembled from over 90 years of intensive antibiotic research and discovery can be leveraged to efficiently select high‐potential antibiotic vector candidates that specifically target the well‐characterized, conserved, and essential molecular pathways of bacteria.^[89]^ For instance, with regards to FDA‐approved antibiotics, information of value to vector selection and radiotracer design is readily available. The most important information is highlighted in Figures 2, 3, 4. This includes a detailed description of the antibiotic mechanism of action; target enzyme binding characteristics; the activity spectrum; pharmacokinetic parameters in general, such as in vivo stability, plasma protein binding, biological half‐life and organ compartment biodistribution patterns; pharmacodynamic parameters such as off‐target host enzyme interactions and drug metabolism pathways; and extensively researched and well defined antibiotic resistance mechanisms (on a molecular and genetic level) that were encountered in clinical isolates over several decades.[^89^, ^93^, ^94^] On this note, well‐established synthesis and quality control methods that adhere to GMP guidelines may be relevant to radiotracer production.[^89^, ^94^] In addition, commercial organizations such as Drugbank (https://go.drugbank.com/), BioHarmony (https://www.biometadata.com/), and Cortellis Clarivate (https://clarivate.com/) provide comprehensive libraries that contain data on antibiotic derivatives that have been disregarded from drug development pipelines; yet, they may still have desirable properties for radiotracer development. The input into radiotracer design that these libraries may provide includes information on SAR and in vitro target binding efficacy. Should the antibiotic candidate drug in question have advanced far enough along the development pipeline, data regarding its in vivo efficacy and biodistribution parameters may also be accessible.

It is worth mentioning that these available datasets may be incorporated into emerging computer‐aided radiopharmaceutical‐design strategies, thereby offering a more evidence‐based, scientific approach. The most useful in silico drug‐design strategies that are transferable to radiopharmaceutical design are: i) a ligand‐based approach (LBDD) using pharmacophore modeling; ii) a structure‐based design approach (SBDD) using molecular docking strategies; and iii) absorption‐distribution‐metabolism‐excretion‐toxicity (ADMET) predictions.[^81^, ^95^] Lastly, radiolabeling an FDA‐approved antibiotic without altering its structure may be subject to FDA pre‐approval and could accelerate clinical translation, along with saving time, money, and resources.

#### 4.4.1 How can a Lack of Antibiotic‐Based Tracer Sensitivity be Addressed?

Antibiotics exert their action by disrupting essential bacterial molecular processes that are moderately expressed and tightly regulated, and thus more easily overwhelmed or saturated by inhibitors.^[89]^ Because of this, and the general low sensitivity (or T/NT) experienced with antibiotic tracers thus far, there are concerns about whether antibiotic‐derived tracers may be too potent to accumulate within bacteria in order to generate a sufficient signal.[^13^, ^72^] However, this theory and its possible influence on signal generation has not been investigated. Therefore, we recommend an experimental strategy like that used for saturable receptor‐targeting radiotracer development (Figure 2). This approach's hypothesis is based on uptake relying on enzyme binding/inhibition and saturation, rather than bulk metabolic turnover or surface adhesion. From this perspective, the main developmental concern is whether the target protein/receptor density (*B*_max_) will sufficiently match the amount of radioactivity in the region of interest (ROI) to generate an appreciable target‐to‐background signal.^[96]^ Secondly, the radiotracer's binding affinity (*K*_d_ or *K*_i_) should be compatible with *B*_max_ since a lower target density will require a stronger‐binding radiotracer at a lower dose or concentration (Figure 2A). Conversely, a radiotracer with a weaker binding affinity will require a higher target density to achieve the same result. A common measurement used to describe this relationship is *B*_max_/*K*_d_, with a value >10 to be considered for prospective receptor‐radiotracer development.[^96^, ^97^, ^98^, ^99^] As an example, this relationship for TMP is several fold above this range when calculated with available *B*_max_ and *K*_d_ values reported in the literature (*B*_max_≈50 nM, *K*_d_<1.3 nM for TMP and *K*_d_<0.465 nM for [^18^F]FP‐TMP).[^16^, ^18^, ^100^] This indicates that the performance of some antibiotics may not be limited by antibiotic potency/underwhelming target protein expression levels. In addition, this indicates that perhaps receptor‐targeting radiotracer development strategies (and its applications) may be translatable towards antibiotic‐PET infection imaging.

Because most antibiotics are characterized by a saturable binding mechanism, one should consider that the tracer mass (extra carrier‐added amount), as part of the radioactive dose, may compete for and partially saturate target binding sites, especially if the number of such targets is limited (Figure 2B). This may significantly influence its binding pharmacokinetics and, ultimately, impair detection sensitivity and outcomes in imaging studies evaluating tracer performance. Usually, in human studies, the mass effect is considered negligible due to the minute quantity of tracer administered (picogram to microgram) relative to the subject mass.^[98]^ However, in small animal PET imaging, this “mass effect” is known to be more prominent due to the higher activity required (based on mCi kg^−1^ bodyweight) to compensate for the smaller subject size and difference in signal attenuation.[^96^, ^97^] It is worth mentioning that, given the concerns over the generally low sensitivity for detecting infections with antibiotic tracers in small animal models thus far, no detailed studies have been done to fully characterize to what extent the mass effect might influence tracer performance.

On a more technical note, concerning the radiopharmaceutical production, the effect of the relationship between specific activity and the administered drug concentration (carrier added/bolus injection) on antibiotic‐derived radiotracer performance is still unknown. Future studies elucidating antibiotic–target relationship mechanics at microdose levels, and standardization of specific activities should prove beneficial for radiotracer development. To illustrate this, radiolabeled ciprofloxacin showed discordant results in animal models with specific activities achieved for both [^68^Ga]‐1/[^68^Ga]‐2 and [^18^F]F16, that were of two and four orders of magnitude higher than that of [^99m^Tc]Tc‐/[^18^F]F‐ciprofloxacin (Table 1).[^26^, ^27^, ^28^, ^34^] Altogether, characterizing the influence of these variable parameters on tracer sensitivity may rationalize future antibiotic candidate selection and guide the determination of optimal tracer dosage.

#### 4.4.2 What are the Challenges Related to the Antibiotic MoA?

Enzyme–drug interaction characteristics and the bactericidal MoA of antibiotics should be considered before infection‐specific radiotracer development (Figure 3). For instance, in 1996, Tewson et al. mentioned a mismatch in using fluoroquinolones as infection imaging agents,^[31]^ based on the fact that two simultaneously‐bound fluoroquinolone molecules are required to form a tight but reversible (noncovalent) inhibitory complex.^[23]^ Since only trace amounts of radiolabeled fluoroquinolone are used, it is highly unlikely to achieve an appreciable amount of inhibitor–enzyme complex formation to generate an adequate signal at the infection site. This is in accordance with the unsaturable and reversible binding nature experienced with fluoroquinolones. Therefore, a suitable antibiotic candidate should preferably bind to the enzyme target without requiring additional ligands since the lack of these within the pathogen environment will probably affect tracer uptake. In the case of an uncompetitive inhibition process, knowledge of the enzymatic substrate formation rate is necessary. Another scenario is the requirement for prodrug activation by some antibiotic classes, such as INH and PZA.

The involvement of a second activator enzyme, in addition to the target protein, may further confound tracer uptake as any modification to the antibiotic may disrupt prodrug conversion and/or target binding, especially at microdose scale.

For instance, radio‐fluorination of 5‐[^18^F]F‐PZA completely diminishes PZAase enzyme recognition and loss of prodrug activation to 5‐[^18^F]F‐POA, resulting in a complete loss of specific uptake.^[61]^ Lastly, some antibiotics (such as INH) become metabolically trapped within the target pathogen through prodrug activation, which is rather beneficial to increase tracer uptake at the target.^[42]^ The puzzling results gathered from fluoroquinolone and PZA tracer development made it clear that the antibiotic MoA should be well known before selecting a potential candidate for subsequent radiolabeling.

#### 4.4.3 Are We Considering the Emergence of Antibiotic Resistance?

With the rapid emergence of complex drug‐resistant bacterial infections, accurate and timely detection of these pathogens is crucial for patient care. An antibiotic tracer significantly affected by antibiotic resistance mechanisms will probably be clinically irrelevant. Resistance mechanisms include, but are not limited to, significant changes in target protein binding and expression levels; the presence of drug exclusion mechanisms such as export pumps; the formation of biofilms, and diminished active transport; and drug inactivation/degradation mechanisms.[^23^, ^69^, ^78^, ^84^, ^88^, ^93^] While overcoming these scenarios with an antibiotic‐derived tracer may seem challenging, promising results have been obtained with [^11^C]TMP to image a wide variety of pathogens, regardless of their resistance status.^[20]^ For instance, [^11^C]TMP uptake was maintained by various strains harboring mutant TMP‐resistant DHFR proteins due to the maintained co‐expression of wild‐type (WT) DHFR. Further analysis of the National Centre for Biotechnology Information's Reference Sequence Collection showed that 99.4 % of TMP‐resistant strains co‐express WT DHFR in addition to its mutant homologue. Interestingly, elevated levels of tracer uptake were also noted in antibiotic‐resistant strains, which were confirmed to express drug export pumps, even notoriously multi‐drug resistant *P. aeruginosa* strains. Similarly, [^18^F]FP‐TMP accumulated in TMP‐resistant *P. aeruginosa*.

#### 4.4.4 Would Antibiotic‐PET Imaging Readouts be Affected by the Empirical Use of Antibiotics?

It is evident that pre‐dosing a system with an antibiotic that competes with a tracer for the same target binding site will result in lower tracer uptake.[^18^, ^19^, ^21^, ^101^] Additionally, treatment with, or co‐administration of, bacteriostatic antibiotics may completely inhibit the expression of the target required for tracer binding, or display some form of synergistic or antagonistic effect resulting in diminished tracer uptake.^[76]^ Thus far, no studies have explored the possible effects of co‐treatment with a different antibiotic class on pathogen‐specific radiotracer accumulation. This is extremely important since most patients referred for PET imaging to detect an underlying infection have often received some form of antibiotic treatment. In this regard, antibiotics that enter the cell through unique MoAs should be prioritized for labeling to avoid the risks associated with bacterial resistance regarding more common MoAs. Given such a widespread empirical and often prophylactic administration of antibiotics to patients with suspected infection, the recruitment of patients with limited or no exposure to antibiotics at the time of PET imaging presents a tremendous challenge.^[72]^

#### 4.4.5 How Can We Balance Tracer Bioavailability Against its Unwanted Biodistribution?

High tracer stability (chemical and physiological) is a main prerequisite for bacterial tracer uptake because it improves bioavailability: i.e., sufficient tracer amount reaches the tissue in which the pathogens reside. In this regard, it is essential to leverage existing antibiotic data with detailed and complete descriptions of experimental ADME properties, as well as important human enzyme and tissue‐specific interactions for optimal candidate selection (Figure 4).^[89]^ For instance, an antibiotic that is known to be associated with the host inflammatory response, such as binding to leukocytes, should be disregarded. Stability assessment of a radiolabeled antibiotic can be performed by mimicking the in vivo environment, such as human plasma stability and protein binding.[^9^, ^71^] In the case of limited stability or high affinity to plasma proteins, its translation potential is doubtful. Furthermore, radionuclides must be incorporated into a position that is not cleaved or modified by host enzymes, especially in the case of prodrug activation. The lack of prodrug activation, growth inhibition and tracer uptake, in addition to a lack of in vivo stability, experienced with 5‐  [^18^F]F‐PZA is a prime example of how unnecessary exhaustive in vivo experiments may have been avoided through preliminary in vitro assessment.^[61]^

Clinically approved antibiotics are generally designed to have relatively long biological half‐lives for sustained therapeutic effect, but for imaging purposes the tracer is required to delineate the target that may be very dispersed or expressed at low levels relative to the surrounding mammalian tissue targets. As a result, when it comes to antibiotic‐derived PET tracers, inherently high plasma concentration and blood perfusion are responsible for a slow and insufficient tissue background clearance, which is a major concern, given the relatively short half‐lives of most used PET radionuclides. Although calculated biological half‐lives of antibiotics are reported to be typically 3 to 12 hours, this does not fully represent individual tissue‐specific compartment half‐lives. Many of the antibiotic tracers presented here are cleared relatively quickly from most peripheral tissues such as the brain, muscle, lungs, vasculature, and bone (which are common sites of bacterial infiltration). Yet, prolonged residence times are seen in the kidneys and/or liver until metabolization and elimination. The latter encourages imaging of infectious processes, such as osteomyelitis, endocarditis, and soft tissue infection, in the extra‐abdominal cavity. Moreover, high and non‐specific organ retention might be of concern in terms of radiation dosimetry. Therefore, approved antibiotics with shorter biological half‐lives in line with the half‐life of the radionuclide should be considered for radiolabeling.

Particular challenges to TB infection imaging hinge on the potential of the drug or imaging agent to tackle the slow growth rate of *M. tuberculosis*, the intricacy of its different disease states and the penetration into the granulomatous lesions in vivo. PK/PD studies using [^11^C]RIF‐, [^18^F]F‐linezolid‐ and [^76^Br]Br‐BDQ‐PET have suggested that intralesional bioavailability of the radiotracers is dependent on both drug characteristics and host factors, such as lesion pathology.[^67^, ^70^, ^71^, ^80^, ^81^, ^87^] Often, blood perfusion of the tissue affects the signal measured for a single time point investigation; therefore, a more complex (kinetic) analysis may be the better strategy. It must also be emphasized that animal models must be used that closely resemble human disease presentation. For example, TB is known to form well‐defined cerebral and pulmonary lesions, often accompanied by cavitating granulomas that are resistant to antibiotic penetration. As such, a rabbit model that develops these lesions when infected with *M. tuberculosis* was chosen to measure intralesional [^11^C]RIF bioavailability.[^81^, ^102^] A strategy that may alleviate these antibiotic‐inherent issues with biodistribution and bioavailability is the incorporation of a radiolabeled functional group, which may significantly alter the physicochemical properties of the desired antibiotic vector, while keeping the pharmacophore intact. Examples of such functionalities are metal chelator conjugates that are highly polar, or incorporation of a radionuclide into an antibiotic via a highly lipophilic conjugate, such as carbon chain spacers.^[8]^ Additionally, drug delivery systems such as liposomes, nanoparticles and microspheres have previously been utilized in nuclear medicine to improve tracer bioavailability and stability.^[103]^ [^18^F]FP‐TMP is a prime example of how minor structural modifications allow for inclusion of a more matching radionuclide that provides better properties for tracing cerebral tracer clearance and thereby also significantly improving imaging sensitivity in comparison to [^11^C]TMP.^[104]^

### 4.5 Future Potential

**Figure 5:**
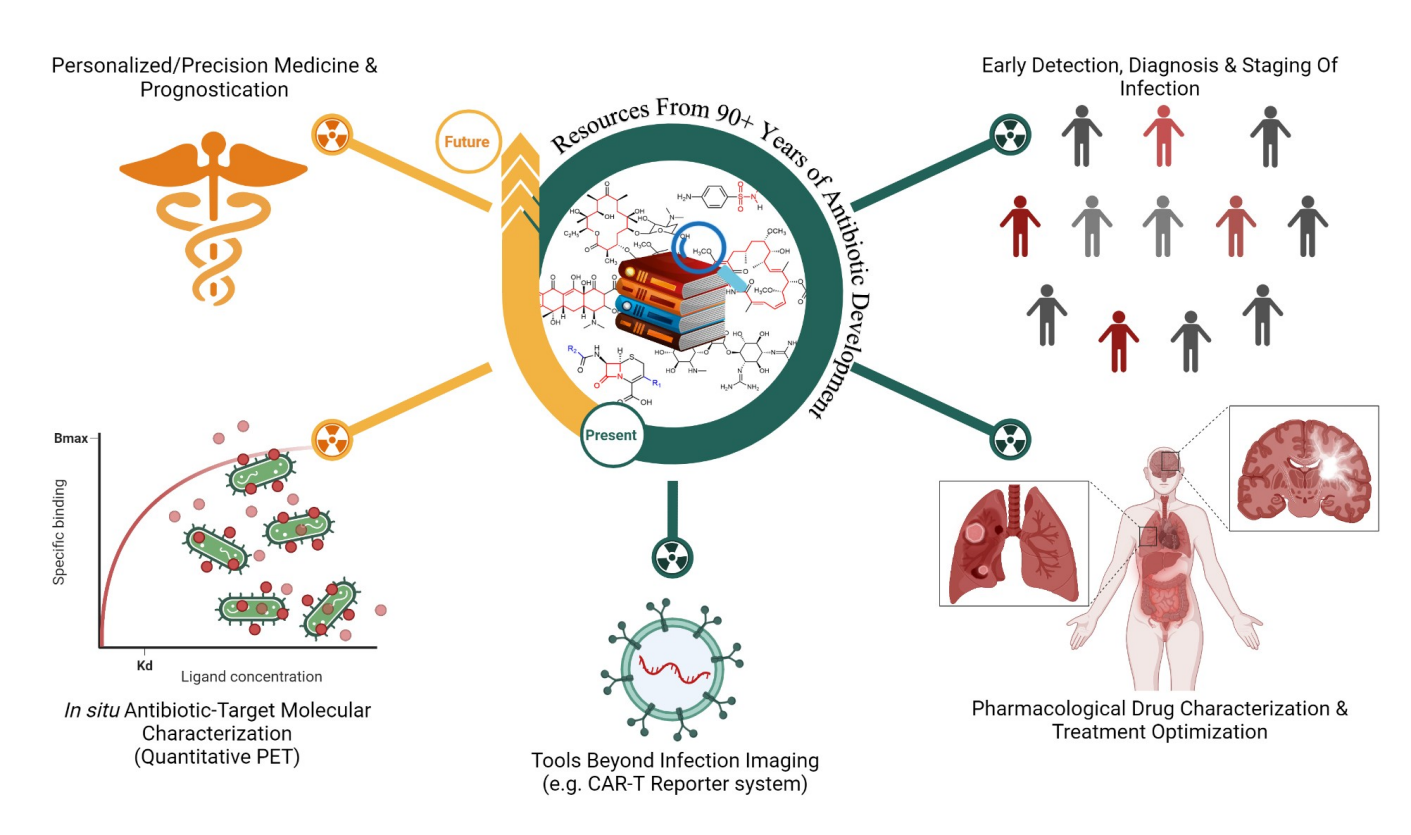
Current and potential applications of radiolabeled antibiotics. Created with Biorender.com.

Future endeavors should focus beyond diagnosing infection and move towards prognostication: to predicting response to treatment; identifying resistance mechanisms; and identifying patients at high risk for complicated/resistant infection (Figure 5).[^13^, ^90^] These goals could (in theory) be achieved by using PET's capabilities to quantitatively monitor and assess molecular pathway activities that characterize and govern the pathophysiological process of infectious diseases, and the interaction of such molecular pathways with treatment interventions in situ. In this regard, antibiotic‐based radiotracers should (in theory) be of value, since the administration of antibiotics is the core subject that governs these issues.

Additionally, because antibiotics follow a specific and saturable binding mechanism, developing an antibiotic radiotracer that can measure essential bacterial enzymes’ ‘binding potential’ may be possible in the future. Measuring an enzyme/receptor's binding potential affords insight into the status of the target protein's rates of expression and activity (not total expression).^[97]^ In conjunction with parallel imaging (two radiopharmaceuticals simultaneously, or paramagnetic tracers for dual‐modality PET/MRI), antibiotic radiotracers may offer yet more opportunities to advance our understanding of the mechanisms governing antibiotic performance and the emergence of resistance.^[90]^ Antibiotic‐PET imaging may also provide valuable input to fine‐tune in silico modeling and drug‐design strategies,[^81^, ^95^] and aid in evaluating the performance of drug‐delivery systems.^[103]^ Antibiotics may find yet more applications beyond infection imaging; Sellmyer et al. proposed the use of *Ec dhfr* as a reporter gene inserted into chimeric antigen receptor (CAR) T cells, and subsequently tracking the CAR T cell distribution using [^18^F]FP‐TMP‐PET to better understand cell trafficking in vivo for modified cell‐based therapies.[^21^, ^101^, ^104^]

## 5 Conclusion

Confidence has waned in antibiotic‐based radiotracers as infection imaging agents due to a history of discouraging results that do not accurately reflect the clinical potential of radiolabeled antibiotics in becoming bacterial‐specific PET imaging agents. We presented critically evaluated literature on antibiotic‐derived PET radiopharmaceutical development efforts aimed at infection imaging. To address pitfalls for clinical translation, profound understanding of the antibiotic MoA, attention to structural tracer design, virtuous pre‐clinical study design, and accurate data acquisition methods are necessary to realize the true potential of these agents as infection‐specific radiopharmaceuticals. As such, [^11^C]TMP and [^18^F]FP‐TMP have sparked renewed interest as infection imaging agents that may diagnose and monitor disease progression, while the value of [^11^C]RIF‐PET in using antibiotics as research tools to monitor in situ PK/PD parameters for the optimization of antibiotic treatment regimens was revealed. Taken together, antibiotic‐derived radiotracers may open the door to precision medicine and more personalized patient care, given that [^11^C]TMP has already proved its capability in monitoring disease progression in patients, and [^11^C]RIF biodistribution has revealed limited intralesional penetration at standard dosage. Antibiotic‐derived radiotracers may promise better tools for disease prognostication and advance our understanding of the mechanisms governing antibiotic performance and the emergence of resistance in the future.

## Conflict of interest

The authors declare no conflict of interest.

## Biographical Information

*Christiaan A. (Arno) Gouws is currently a Ph.D. candidate under the joint supervision of Prof. Tricia Naicker and Prof. Thomas Ebenhan at the Catalysis and Peptide Research Unit (CPRU), University of KwaZulu‐Natal. He received his BSc in Microbiology and Biochemistry in 2014, and BSc (Hon) in Biochemistry in 2015 from North West University, Potchefstroom. He completed his MSc in Pharmaceutical Chemistry in 2018 at CPRU under the supervision of Prof. Thavendran Govender. During this period, he worked on the design, synthesis, and preliminary evaluation of potential infection‐specific radiotracers. His current research interests include the design, preliminary evaluation and novel applications of infection‐specific radiotracers to help address the everlasting threat of infectious diseases*.

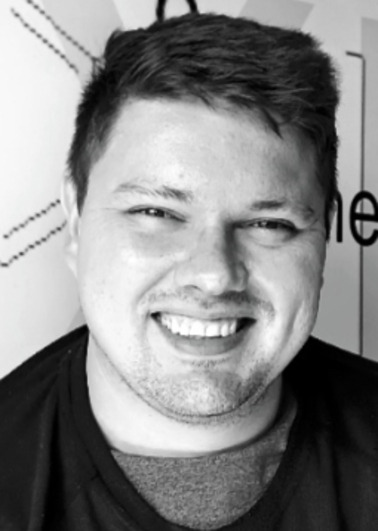

## Biographical Information

*Gert (H. G.) Kruger graduated from Potchefstroom University, South Africa, in 1996 under the supervision of Frans (F. J. C.) Martins and Attie (A. M.) Viljoen. His Ph.D. lineage is traced back to Rudolf Criegee (Würzburg) via Johan Dekker (Karlsruhe). The Dekkers introduced cage chemistry to South Africa, and Kruger actively pursues the synthesis, computational chemistry, and biotesting of cage compounds in the Catalysis and Peptide Research Unit based at the University of KwaZulu‐Natal*.

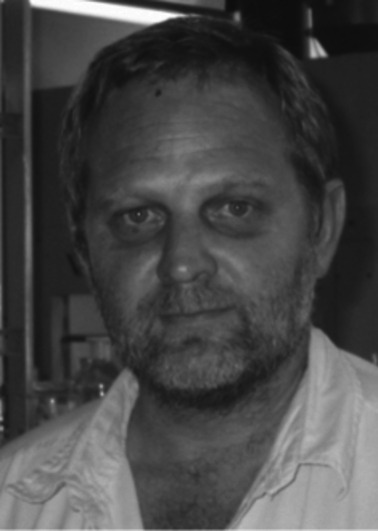

## Biographical Information

*Olivier Gheysens is Deputy Head at the Department of Nuclear Medicine, Cliniques Universitaires Saint‐Luc Brussels and is a member of the Institute of Experimental and Clinical Research at the Université Catholique de Louvain (Belgium). He is chair of the Inflammation and Infection committee of the European Association of Nuclear Medicine (EANM). His clinical and research expert areas are non‐invasive multimodal imaging of cardiovascular disorders, infectious and inflammatory disorders as well as hematologic malignancies. He has been involved in writing several European guidelines and procedural recommendations for nuclear imaging in infectious and inflammatory disorders*.

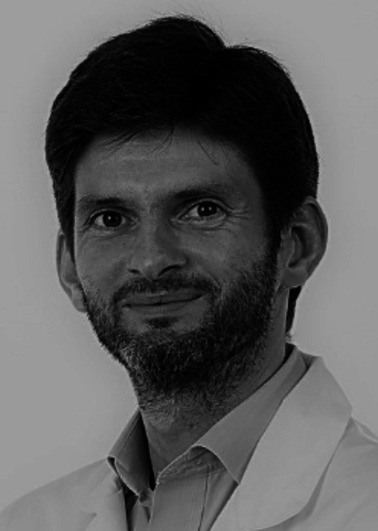

## Biographical Information

*Jan Rijn. Zeevaart received his PhD from the Techniese Universiteit Delft in the Netherlands in 2001. In 2002 he was appointed as head of the Radiochemistry group at the Nuclear Energy Corporation of South Africa. He is a NRF rated researcher since 2009 with a current rating of B2. He has been involved in the development of the isotope production processes that now reside with NTP Radioisotopes SOC Ltd. These commercialization efforts earn South Africa more than 50 MR per annum in foreign revenue. His research disciplines are radioisotope production and radiopharmaceutical chemistry, and he has a keen interest in exploiting these disciplines in designing new radiopharmaceuticals. Zeevaart has been appointed an Extraordinary Professor at both North West University and University of Pretoria*.

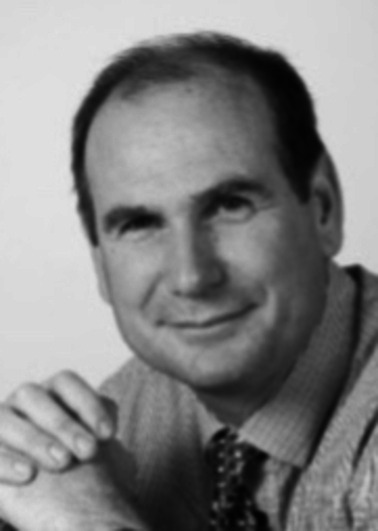

## Biographical Information

*Thavendran Govender graduated from the University of KwaZulu‐Natal South Africa in 2005 under the supervision of Hendrik G. Kruger and Glenn E. M. Maguire. He undertook postdoctoral studies at Uppsala University, in Sweden, with Prof. P. I. Arvidsson. In 2007, he became a lecturer at the University of KwaZulu‐Natal. He was the founding director and principal investigator at the Catalysis and Peptide Research Unit. He is currently a Professor at the University of Zululand*.

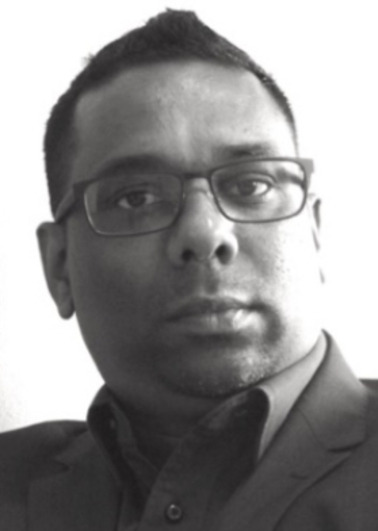

## Biographical Information

*Tricia Naicker received her M.Sc. (cum laude) from the University of KwaZulu‐Natal in 2008 for her work in asymmetric catalysis. In 2011, she completed her Ph.D. at the same institution, working on developing and applying novel tetrahydroisoquinoline‐based organocatalysts. Afterward, she joined Prof. K. A. Jørgensen's research group at the Centre for Catalysis at Aarhus University, Denmark, as a postdoctoral fellow. Since 2013, she started her academic career and independent research endeavours at the Catalysis and Peptide Research Unit based at UKZN. Currently she serves as the vice‐chair for the Organisation for Women in Science for the Developing World (National Chapter) and she has been inaugurated into the South African Young Academy of Science*.

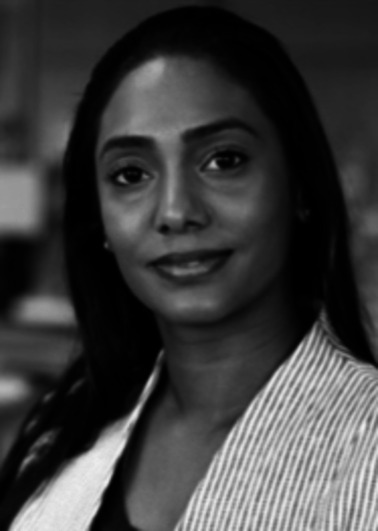

## Biographical Information

*Prof. Thomas Ebenhan is trained biochemist and pharmacologist from the University of Tübingen, Germany featuring a 16‐year academic record combined with a 3 year insert of corporate involvement at Novartis Pharma AG, Switzerland. He holds an Associate Professorship at University of Pretoria, is managing a preclinical molecular imaging facility and is involved in the development of a unique nuclear medicine research infrastructure development for South Africa. He completes duties as chief investigator, as scientific advisor/expert or as student (co‐) supervisor on multi‐institutional, multi‐scientific projects, mostly in biomedical or natural sciences*.